# Herbicidal Activity and Molecular Docking Study of Novel ACCase Inhibitors

**DOI:** 10.3389/fpls.2018.01850

**Published:** 2018-12-18

**Authors:** Fei Ye, Peng Ma, Yuan-Yuan Zhang, Ping Li, Fei Yang, Ying Fu

**Affiliations:** Department of Applied Chemistry, College of Science, Northeast Agricultural University, Harbin, China

**Keywords:** 2-phenyl-3-cyclohexenone, design, synthesis, ACCase, herbicidal activity, molecular docking

## Abstract

Acetyl-CoA carboxylase (ACCase) is an important target enzyme for the development of new bleaching herbicides. On the basis of structure-activity relationships and active subunit combinations, a series of novel 2-phenyl-3-cyclohexanedione enol ester derivatives was designed and synthesized by coupling and acylation reactions. The preliminary biological tests indicated good post-emergent herbicidal activity at a dosage of 150–300 g ai/ha, superior to that of clethodim against barnyard grass. Compound **3d** was safe with respect to maize, even at a dosage of 300 g ai/ha. Compound **3d** showed the best ACCase inhibitory activity *in vitro*, with a value of 0.061 nmol h^-1^ mg^-1^ protein, superior to that of clethodim. Molecular docking modeling showed that compound **3d** and clethodim had the same interactions with surrounding residues, leading to an excellent combination with the active pocket of ACCase. That may have been the mechanism responsible for the death of the barnyard grass. The present work suggests compound **3d** as a potential lead structure for further development of novel ACCase inhibitors.

## Introduction

Acetyl-CoA carboxylase (ACCase, EC 6.4.1.2) performs the first and crucial step in fatty acid biosynthesis and has been found in most biological organisms, including bacteria, fungi, plants, and humans and other animals (Wan et al., 2004; Huerlimann and Heimann, 2013; Keereetaweep et al., 2018; Tomassetti et al., 2018). ACCase catalyzes the formation of malonyl-CoA, which has a vital role in the biosynthesis of long chain fatty acids for the maintenance of cell functions (Joyard et al., 2010; Yang et al., 2018). The production of malonyl CoA takes place in two stages, catalyzed by ACCase, starting with acetyl-CoA and CO_2_. First, the biotin carboxylase subunit of ACCase catalyzes the ATP-dependent carboxylation of biotin. Next, the carboxyltransferase subunit catalyzes the transport of an activated group containing carboxyl to its acceptor acetyl-CoA (Xiang et al., 2009). The plant becomes bleached and eventually undergoes necrosis and death when ACCase is inhibited. Herbicides targeting ACCase have the advantages of a wide weed-control spectrum, flexible application time, and well compatibility with other herbicides (Powles, 2005; Tehranchian et al., 2017; Lancaster et al., 2018). However, the frequent use of this class of herbicides has resulted in increasing resistance in many grass weeds (Laforest et al., 2017; Mccullough et al., 2017; Saini et al., 2017). As a consequence, there is commercial demand for the development of new herbicides that inhibit ACCase in both the susceptible and resistant forms of plants (Shukla et al., 2004).

**FIGURE 1:**
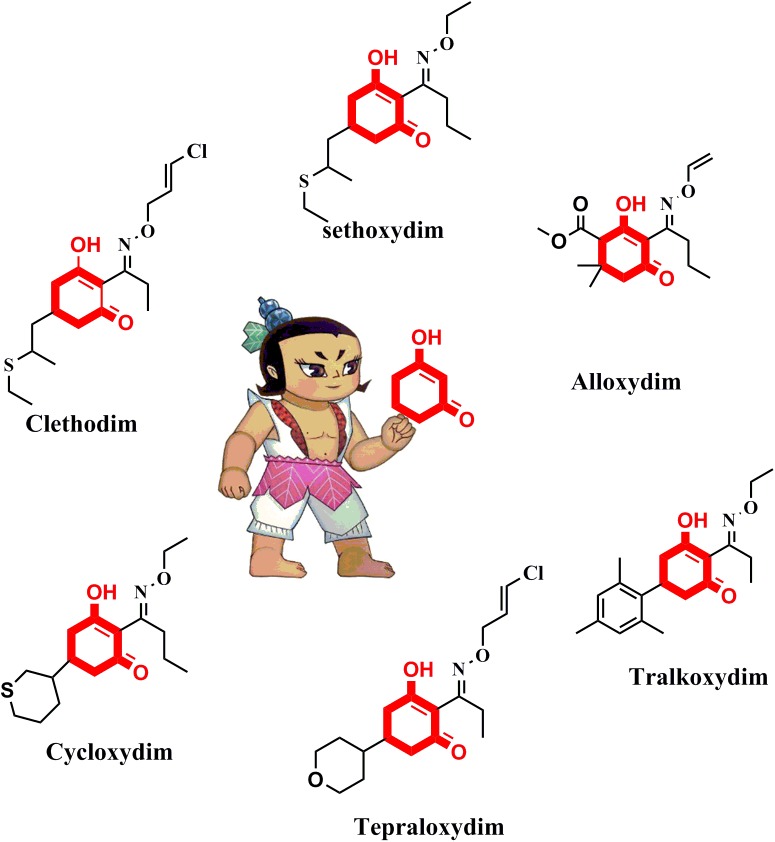
Chemical structures of the commercial CHD inhibitors.

The active ingredients of ACCase inhibitors are classified into the aryloxyphenoxypropionate (FOP), cyclohexanedione (CHD), and phenylpyrazoline (DEN) chemical families (Shergill et al., 2016). CHD derivatives represent a very active research area owing to their extensive structural diversity (Seng et al., 2003; Louie et al., 2010). As shown in Figure 1, there are six frequently used commercial CHD inhibitors: clethodim, sethoxydim, alloxydim, cycloxydim, tepraloxydim, and tralkoxydim. The common chemical motif of CHD inhibitors is the 1,3-cyclohexanedione, which forms the minimum substructure (Webb et al., 2000; Oh et al., 2015; Smejkal et al., 2017). An extensive review of the literature on ACCase inhibitors has suggested that modification of the cyclohexanedione portion is a feasible approach to develop new derivatives with improved herbicidal activity.

**FIGURE 2:**
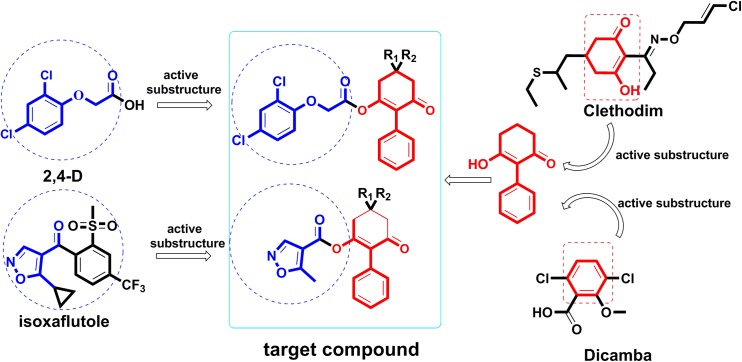
Design of the target compounds.

Thus, attempts to design novel compounds targeting ACCase by keeping the cyclohexanedione subunit as the parent skeleton should prove valuable for solving the problem of resistance. As part of our ongoing work (Ye et al., 2018), herein we report the design of a series of novel 2-phenyl-3-cyclohexanedione enol ester derivatives, based on structure-activity relationships (SAR) and active subunit combinations (Figure 2).

## Materials and Methods

### Instruments and Materials

Infrared (IR) spectra were taken on an ALPHA-T infrared spectrophotometer using KBr disks. The ^1^H nuclear magnetic resonance (NMR) and ^13^C NMR spectra were recorded on a Bruker AVANCE 400 MHz, with CDCl_3_ as the solvent and tetramethylsilane (TMS) as the internal standard. The elemental analysis was performed on a FLASH EA1112 elemental analyzer. The mass spectrum was recorded on a Waters Xevo TQ spectrometer. X-ray diffraction data were collected on a Bruker AXS II CCD area-detector diffractometer, Mo *K*α. The melting points were determined on a Beijing Taike melting point apparatus (X-4) and are uncorrected. Microwave experiments were carried out with a reliable microwave reactor (Beijing XH-100A) at 800 W. The kits (Shanghai Generay Biotech Co., Ltd., Beijing, China) were commercially available and prepared with standard methods before use. All reagents were of analytical grade.

### General Synthetic Procedure for 3-Hydroxy-2-Phenylcyclohex-2-En-1-One Derivatives 2

Iodobenzene (1.02 g, 5 mmol) was added dropwise to a solution of substituted 1,3-cyclohexanedione **1** (15 mmol) in DMSO, with *L*-proline and CuI as the catalyst, and anhydrous K_2_CO_3_ as the acid-binding agent. The mixture was refluxed for 40 min under microwave irradiation (800 W). The organic phase was dried over anhydrous Na_2_SO_4_ and the solvent was removed by vacuum distillation. The crude product was purified by column chromatography with petroleum ether and EtOAc (V:V = 3:1) as the eluent, or by recrystallization in EtOAc-n-hexane (in the case of solid ethers). The spectral data for intermediates **2a–c** are available in the Data Sheet S1 of Supplementary Material.

### General Synthetic Procedure for 2-Phenyl-3-Cyclohexanedione Enol Ester Derivative 3

A series of 3-hydroxy-5,5-dimethyl-2-phenylcyclohex-2-en-1-one derivatives **2** were mixed with acyl chloride (1.2 mmol) in a round-bottomed flask in the presence of Et_3_N (1.5 mmol). The mixture was vigorously stirred at 25°C for 1 h. The reaction mixture was extracted with CH_2_Cl_2_ and filtered. The organic layer was dried over anhydrous Na_2_SO_4_, and CH_2_Cl_2_ was evaporated under vacuum. The crude product was purified by column chromatography with petroleum ether and EtOAc (*V*:*V* = 3:1–9:1) as the eluent, or by recrystallization in EtOAc-n-hexane.

### Compound Data

### 3-[5-Methyl-3-Isoxazole-4-Carbonyloxy]-2-Phenyl-3-Cyclohexen-1-One (3a)

White solid; yield, 55%; mp, 90-91°C; IR (KBr, cm^-1^) *ν*: 3083-2842 (C-H), 1719, 1663 (C = O), 1072 (C-O); ^1^H NMR (300 MHz, CDCl_3_, ppm) *ä*: 8.30 (s, 1H, N = CH), 7.13-7.35 (m, 5H, Ar-H), 2.85-2.88 (t, 2H, J = 9.3 Hz, CH_2_), 2.65-2.69 (t, 2H, J = 9.9 Hz, CH_2_), 2.47(s, 3H, O-C-CH_3_), 2.20-2.27 (m, 2H, CHi_2_);^13^C NMR (75 MHz, CDCl_3_, ppm) *ä*: 197.62, 175.47, 164.39, 158.15, 150.00, 131.24, 130.72, 129.46, 129.46, 128.06, 128.06, 127.98, 108.40, 37.55, 29.12, 20.88, 12.48. High-resolution mass spectrometry (HRMS) [electrospray ionization (ESI)]: *m/z* [M+Na]^+^ calculated for monoisotopic mass 297.1001, found 320.0893.

### 3-[5-Methyl-3-Isoxazole-4-Carbonyloxy]-2-Phenyl-5-Methyl-3-Cyclohexen-1-One (3b)

White solid; yield, 62%; mp, 92-93°C; IR (KBr, cm^-1^) *ν*:3073-2850 (C-H), 1728, 1652 (C = O), 1070 (C-O);^1^H NMR (300 MHz, CDCl_3_, ppm) *ä*: 8.31 (s, 1H, N = CH), 7.12-7.35 (m, 5H, Ar-H), 2.66-2.83 (m, 2H, CH_2_), 2.5-2.56 (m, 1H, CH), 2.48 (s, 3H, O-C-CH_3_), 2.35-2.42 (m, 2H, CH_2_), 1.21-1.23 (d, 3H, J = 9.6 Hz, CH_3_);^13^C NMR (75 MHz, CDCl_3_, ppm) *ä*: 197.63, 175.51, 163.76, 158.21, 149.99, 131.17, 130.25, 129.46, 129.46, 128.07, 128.07, 127.99, 108.40, 45.79, 37.26, 28.66, 20.91, 12.49. HRMS (ESI): *m/z* [M+Na]^+^ calculated for monoisotopic mass 311.1158, found 334.1050.

### 3-[5-Methyl-3-Isoxazole-4-Carbonyloxy]-2-Phenyl-5,5-Dimethyl-3-Cyclohexen-1-One (3c)

White solid; yield, 66%; mp, 98-99°C; IR (KBr, cm^-1^) *ν*: 3081-2850 (C-H), 1722, 1654 (C = O), 1072 (C-O); ^1^H NMR (300 MHz, CDCl_3_, ppm) *ä*: 8.31(s, 1H, N = CH), 7.14-7.35(m, 5H, Ar-H), 2.74 (s, 2H, CH_2_), 2.54 (s, 2H, CH_2_), 2.49 (s, 3H, O-C-CH_3_), 1.26 (s, 6H, CH_3_); ^13^C NMR (75 MHz, CDCl_3_, ppm) *ä*: 197.54, 175.51, 162.66, 158.33, 149.96, 131.09, 129.69, 129.45, 129.45, 128.08, 128.08, 128.00, 108.40, 51.45, 42.92, 32.78, 28.22, 28.22, 12.48. HRMS (ESI): *m/z* [M+Na]^+^ calculated for monoisotopic mass 325.1314, found 348.1206.

### 3-(2,4-Dichlorophenoxyacetyloxy)-2-Phenyl-3-Cyclohexen-1-One (3d)

White solid; yield, 38%; mp, 111–112°C; IR (KBr, cm^-1^) *ν*: 3064-2851 (C-H), 1769, 1665 (C = O), 1140 (C-O); ^1^H NMR (300 MHz, CDCl_3_, ppm) *ä*: 6.06-7.41 (m, 8H, Ar-H), 4.56 (s, 2H, O = C-CH_2_-O), 2.72-2.76 (t, 2H, J = 9.3 Hz, CH_2_), 2.62-2.66 (t, 2H, J = 9.9 Hz, CH_2_), 2.17-2.24 (m, 2H, CH_2_); ^13^C NMR (75 MHz, CDCl_3_, ppm) *ä*: 197.40, 164.98, 163.97, 151.84, 131.08, 131.03, 130.31, 129.70, 129.70, 128.17, 128.17, 128.07, 127.64, 127.25, 124.00, 114.18, 65.71, 37.41, 28.90, 20.52. HRMS (ESI): *m/z* [M+Na]^+^ calculated for monoisotopic mass 390.0426, found 413.0318.

### 3-(2,4-Dichlorophenoxyacetyloxy)-2-Phenyl-5-Methyl-3-Cyclohexen-1-One (3e)

White solid; yield, 43%; mp, 115–116°C; IR (KBr, cm^-1^) *ν*: 3074-2851 (C-H),1768, 1656 (C = O), 1125 (C-O); ^1^H NMR (300 MHz, CDCl_3_, ppm) *ä*: 6.08-7.39 (m, 8H, Ar-H), 4.55-4.57(d, 2H, J = 7.2 Hz, O = C-CH_2_-O), 2.66-2.72 (m, 2H, CH_2_), 2.52-2.59 (t, 1H, CH_2_), 2.48-2.51 (t, 1H, CH), 2.32-2.39 (m, 1H, CH_2_), 1.19-1.20 (d, 3H, J = 9.6 Hz, CH_3_); ^13^C NMR (75 MHz, CDCl_3_, ppm) *ä*: 197.33, 165.11, 163.34, 151.86, 131.02, 130.56, 130.31, 129.68, 129.68, 128.15, 128.15, 128.05, 127.63, 127.27, 124.03, 114.23, 65.73, 45.61, 36.89, 28.28, 20.78. HRMS (ESI): *m/z* [M+Na]^+^ calculated for monoisotopic mass 404.0582, found 427.0474.

### 3-(2,4-Dichlorophenoxyacetyloxy)-2-Phenyl-5,5-Dimethyl-3-Cyclohexen-1-One (3f)

White solid; yield, 50%; mp, 90–91°C; IR (KBr, cm^-1^) *ν*: 3062-2855 (C-H), 1763, 1661 (C = O), 1135 (C-O); ^1^H NMR (300 MHz, CDCl_3_, ppm) *ä*: 6.05-7.40 (m, 8H, Ar-H), 4.56 (s, 2H, O = C-CH_2_-O), 2.62 (s, 2H, CH_2_), 2.51 (s, 2H, CH_2_), 1.22 (s, 6H, CH_3_); ^13^C NMR (75 MHz, CDCl_3_, ppm) *ä*: 197.31, 165.12, 162.40, 151.81, 130.94, 130.31, 129.99, 129.69, 129.69, 128.18, 128.18, 128.07, 127.63, 127.22, 123.96, 114.07, 65.61, 51.32, 42.62, 32.58, 28.18, 18.18. HRMS (ESI): *m/z* [M+Na]+ calculated for monoisotopic mass 418.0739, found 441.0631.

### X-Ray Diffraction

Compound **3a** was recrystallized from EtOAc/n-hexane to afford a suitable single crystal. The cell dimensions and strength of compound **3a** were gauged (298 K) using a Rigaku R-AXIS RAPID area detector diffractometer (Japan) with graphite monochromated Mo-*K*α radiation (λ = 0.71073 Å). 17827 measured reflections and 3651 independent reflections (*R*_int_ = 0.0247) were obtained in the range of 2.78° < 𝜃 < 28.29° (*h*, -14 to 14; *k*, -19 to 19; *l*, -11 to 12), and 2917 observed reflections with *I* > 2*σ* (*I*) were used in the refinement on *F*^2^. The structure was solved by direct methods and refined utilizing SHELXS-97 (Sheldrick, 1997). The crystallographic data have been deposited at the Cambridge Crystallographic Data Centre (CCDC) as supplementary publication number CCDC 1856348. Copies of the data can be obtained, free of charge, upon application to CCDC, 12 Union Road, Cambridge CB2 1EZ, United Kingdom (fax: +44(1223)336033; e-mail: deposit@ccdc.cam.ac.uk).

### Biological Activity Tests

The bioassays of the chlorophyll content of barnyard grass and maize were performed following previously published procedures (Wang et al., 2013). The sprayed dosage of clethodim and synthetic compounds was confirmed through preliminary screening. Barnyard grass was planted in plastic cups and grown in a greenhouse at around 25°C. The spraying treatment was conducted at a dosage of 300 g ai/ha, when the barnyard grass had grown to the four-leaf stage. After 6 days, the *C*_a_ and *C*_b_ contents of the barnyard grass were determined. Each treatment was replicated three times.

The safety of the compounds with respect to maize was also determined. When maize had grown to the two-leaf stage, it was sprayed at the same dosage as the barnyard grass. After 6 days, the *C*_a_ and *C*_b_ contents of maize were determined.

### Determination of ACCase Activity

The barnyard grass and soil were treated in the same way as in the biological activity tests. ACCase was extracted according to the procedures of Cocker et al. (2000), with a few modifications. One gram of barnyard grass leaves was grinded in liquid nitrogen to obtain frozen tissue, which was then homogenized in 10 mL extraction buffer [100 mM tricine (pH = 8), 1 mM ethylenediaminetetraacetic acid, 10% glycerol, 2 mM benzamidine hydrochloride, 0.5% polyvinylpyrrolidone, 20 mM dithiothreitol, and 1 mM phenylmethylsulfonyl fluoride]. The homogenate was filtered and kept on ice until it was centrifuged (25,000 *g*, 4°C, 20 min) to remove cell debris. The supernatant was regulated by 2 mM 40% (NH_4_)_2_SO_4_ and stirred for 30 min (4°C). Afterward, the mixture was centrifuged for 20 min (25,000 *g*). The amount of acetylmethylcarbinol was used to represent the ACCase activity. ACCase activity was assayed according to the kit instructions. Measurements of protein content were obtained following a previously published procedure (Bradford, 1976).

### Computational Methods

The 3D structures of compound **3d** and clethodim were constructed using the sketch module of SYBYL-X 2.0 (Sybyl, 2002, Version 6.9). Subsequently, the Gasteiger-Huckel method was employed to calculate the partial atomic charges, and the molecules were optimized. The crystal structure of ACCase [Protein Data Bank (PDB) ID: 3K8X] was obtained from the PDB. Docking was modeled using the CDOCKER method in Accelrys Discovery Studio 2.5 (Catalyst, 2005, Version 4.10). In the preparation of protein structures, water and some other co-crystallized small molecules were removed, and a CHARMM force field was applied. After that, the binding site was limited to a region within 13.0 Å of the center of the known ligand. Energy minimization performed using smart minimization, and -CDOCKER_ENERGY values were obtained through molecular dynamics simulations. CDOCKER generates random ligand conformations, and a variable number of rigid-body rotations/translations are applied to each conformation to generate the initial ligand poses. The final poses of the ligands were then subjected to simulated annealing to refine the poses of compounds. For each ligand, the top 10 conformations were saved, and the remaining parameters were set to default values.

## Results and Discussion

### Chemistry

**FIGURE 3:**
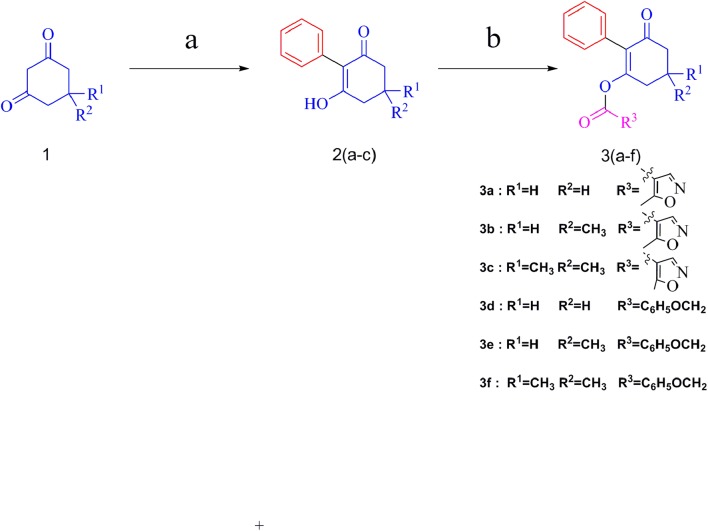
Synthetic route of compounds 3a–f. Reagents and conditions: (a) iodobenzene, K_2_CO_3_, CuI, L-proline, DMSO, microwave irradiation, 800 W, 90°C; (b) 5-methylisoxazole-4-carbonyl chloride or (2,4-dichloro-phenyl)-acetyl chloride, TEA, DCM, rt.

The synthesis of target compounds followed the route outlined in Figure 3. The preparation of compounds **2** involved the coupling reaction of substituted 1,3-cyclohexanedione with iodobenzene in DMSO, and the mixture was refluxed for 40 min under microwave irradiation (800 W) to give yields of 79–88%.

Compounds **3** were obtained by the acylation of compounds **2** with 5-methylisoxazole-4-carbonyl chloride or (2,4-dichloro-phenyl)-acetyl chloride in CH_2_Cl_2_ under stirring for 1 h, with 38-66% yields. The effect of the substituent in the 5-position of cyclohexanedione was explored. Generally, better yields were obtained for compounds with two methyl groups substituted, compared with those with one methyl. For example, the yield of compound **3c**, at 66%, was better than that of compound **3b**. Moreover, the yield of compound **3a**, with no substitute, was lower than that of compound **3b**, with one methyl substitution, which was mainly due to significant effects of the electron-donating group. All the target compounds were confirmed via ^1^H NMR, ^13^C NMR, and HRMS, and the data are reported in the Experimental section.

### Crystal Structure of Compound 3a

**FIGURE 4:**
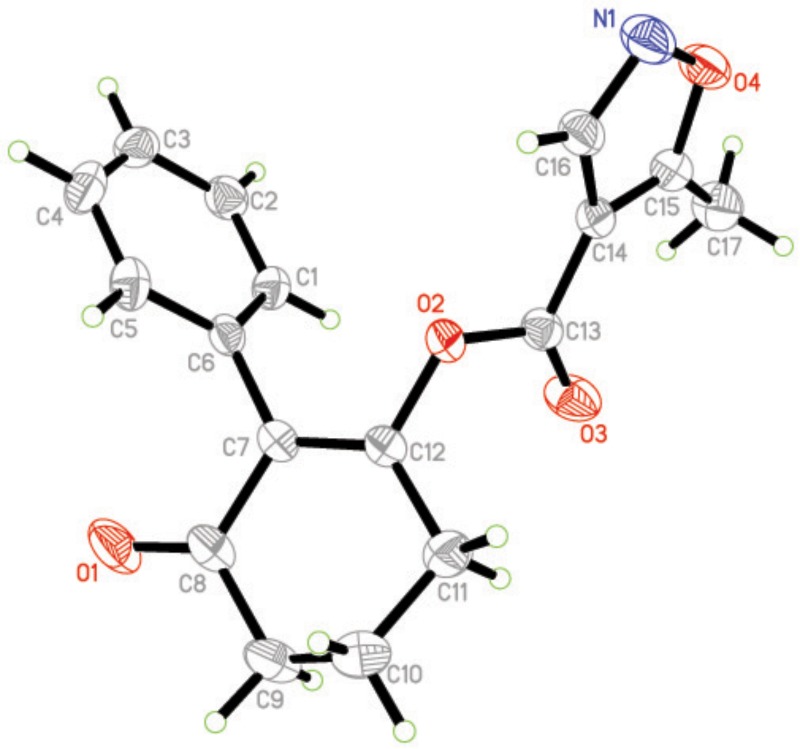
Molecular structure for compound 3a at 30% probability level.

**FIGURE 5:**
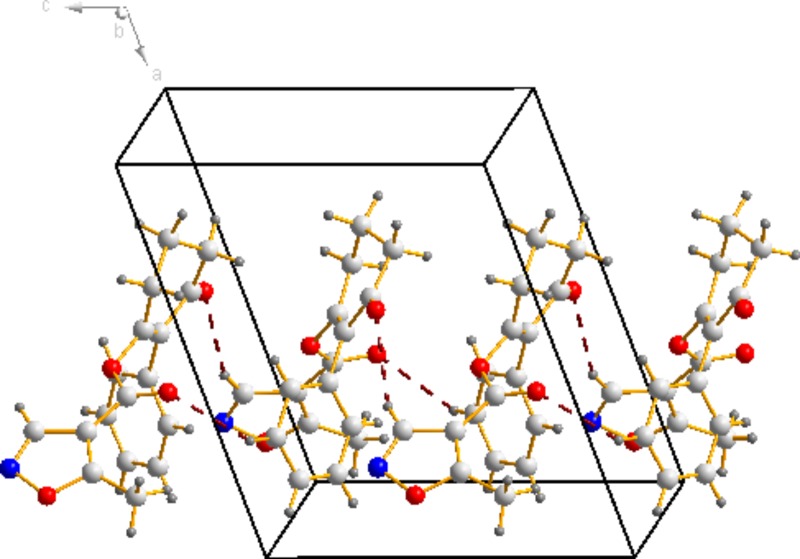
Packing view of the compound 3a.

The molecular structure of compound **3a** is shown in Figure 4 The bond distances of C(12)-O(2) and C(13)-O(2) are 1.399(16) Å and 1.351(19) Å; these are shorter than the normal C-O distance (1.432 Å), indicating a p-π conjunction effect between the carbonyl carbon and the oxygen atom. The dihedral angle between the benzene plane (C1/C1/C3/C4/C5/C6) and the cyclohexanedione plane (C7/C8/C9/C10/C11/C12) is 68.72(53)°. The isoxazole is almost perpendicular to the cyclohexanedione, with a dihedral angle of 85.16(58)°. No significant π-π interaction was found in the crystal structure (Figure 5).

### Biological Activity Tests

The herbicidal activities of compounds **3a–f** (300 g ai/ha) against barnyard grass in the greenhouse experiment are listed in Table 1. The commercial cyclohexanedione herbicide clethodim was chosen as a control. The results for the synthesized compounds showed different degrees of herbicidal activity, representing the unique bleaching symptoms that occurred with declining chlorophyll a (*C*_a_) and chlorophyll b (*C*_b_) content. It is very inspiring that compound **3d** displayed the best herbicidal activity, even better than that of clethodim, while the activities of compounds **3a** and **3e** were similar to that of clethodim. Other compounds, such as **3b**, **3c**, and **3f** were also found to have some degree of herbicidal activity against the tested weed. The SAR revealed marked effects of substituents R^1^ and R^2^ on the bioactivity of the compounds. For example, compounds **3a** and **3d**, with no substituents on the 1,3-cyclohexanedione ring (*R*^1^ = *R*^2^ = H) showed higher activities than the corresponding substituted analogs **3c** and **3f** (*R*^1^ = *R*^2^ = CH_3_), respectively. Moreover, from a structural perspective, the introduction of the substructure of (2,4-dichloro-phenyl)-acetyl resulted in excellent herbicidal activity that led to unique bleaching symptoms for barnyard.

**Table 1:** The chlorophyll content of barnyard grass and maize treated with target compounds^a^.

Compound	Barnyard grass		Maize	
	*C*_a_ (mg/g)	*C*_b_ (mg/g)	*C*_a_ (mg/g)	*C*_b_ (mg/g)
3a	0.882 ± 0.016	0.396 ± 0.017	0.613 ± 0.026	0.256 ± 0.025
3b	1.038 ± 0.010	0.463 ± 0.042	0.686 ± 0.037	0.308 ± 0.025
3c	1.231 ± 0.036	0.509 ± 0.035	0.617 ± 0.027	0.269 ± 0.019
3d	0.627 ± 0.008	0.278 ± 0.056	0.896 ± 0.037	0.251 ± 0.026
3e	0.762 ± 0.023	0.356 ± 0.010	0.469 ± 0.015	0.280 ± 0.033
3f	0.957 ± 0.031	0.561 ± 0.026	0.729 ± 0.018	0.395 ± 0.024
CK	1.404 ± 0.008	0.633 ± 0.021	1.093 ± 0.039	0.295 ± 0.034
Clethodim	0.717 ± 0.010	0.401 ± 0.016	0.527 ± 0.042	0.235 ± 0.034

^a^*Data are means of three replicates*.

Following the herbicidal tests, all compounds were assessed for their safety with respect to maize. In comparison with clethodim, compounds **3d** and **3f** showed better safety. However, the herbicidal activity of compound **3f** was not as good as that of compound **3d**. Therefore, compound **3d** represents a potential lead structure for further development of novel herbicides for weed control in maize.

### ACCase Activity Tests

Cyclohexanedione herbicides are a kind of ACCase inhibitors. The ACCase activities *in vitro* were assayed to verify the herbicide activity of the synthesized compounds (Table 2). According to the results, clethodim led to a marked decline in ACCase activity. The target compounds showed different degrees of inhibition of ACCase activity, ranging from 0.061 to 0.385 nmol h^-1^ mg^-1^ protein. Compound **3d** exhibited the best ACCase inhibitory activity, with a value of 0.061 nmol h^-1^ mg^-1^ protein. Although the compounds **3a**, **3e**, and **3f** were not such potent inhibitors as clethodim, they still showed good ACCase-inhibiting activity, while compounds **3b** and **3c** had lower ACCase inhibitory activity, with values of 0.312 and 0.385 nmol h^-1^ mg^-1^ protein, respectively. From the perspective of SAR, the substituents at the 5 position of the 1,3-cyclohexanedione considerably reduced the ACCase-inhibiting activity. The hydrogen atoms at the 5 position of the 1,3-cyclohexanedione in the target compounds **3a** and **3d** were substituted with one methyl group (**3b**, **3e**) or two methyl groups (**3c**, **3f**) led to decreasing enzyme inhibitory activity, and all these substituted compounds displayed lower activity than **3a** and **3d** (**3a**>**3b**>**3c**, **3d**>**3e**>**3f**). These results indicate that non-polar alkyl substituents at the R^1^ and R^2^ position are unfavorable in terms of ACCase inhibition.

**Table 2:** ACCase-inhibiting activities of compounds **3a-f** (300 g ai/ha^-1^)^a^.

Compound	ACCase activity (nmol h^-1^ mg^-1^ protein)	Compound	ACCase activity (nmol h^-1^ mg^-1^ protein)
3a	0.103 ± 0.016	3e	0.089 ± 0.011
3b	0.312 ± 0.023	3f	0.125 ± 0.009
3c	0.385 ± 0.019	CK	0.681 ± 0.043
3d	0.061 ± 0.004	Clethodim	0.082 ± 0.002

^a^*Data are means of three replicates*.

### Molecular Structure Comparisons

The greenhouse experiments and ACCase activity tests showed that the synthesized compounds displayed different degrees of herbicidal activity. Comparing the physicochemical properties of the synthesized compounds and clethodim, it was notable that the log *p*, hydrogen bond acceptors(HBAs), hydrogen bond donors (HBAs), molecular weight (MW) and surface area of compounds **3c** and **3d** were all similar to those of clethodim, but the -CDOCKER ENERGY of compound **3d** was significantly higher than that of compound **3c** (Table 3). This indicates that, based on its physicochemical properties, compound **3d** has potential as a lead structure for further development of novel ACCase inhibitors.

**Table 3:** Comparison of physicochemical properties of synthesized compounds and clethodim.

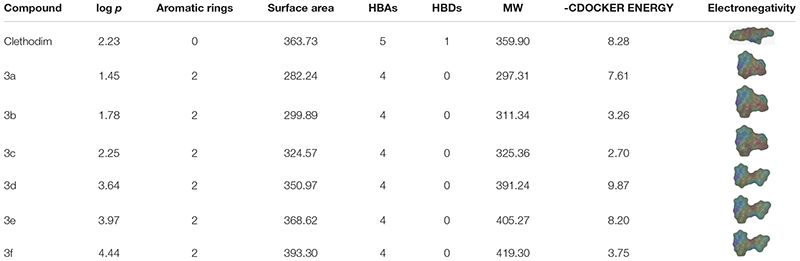

*Predicted values were obtained using ChemBioOffice 2016 for log p, aromatic rings, and molecular weight (MW); Discovery Studio 2.5 for surface area, -CDOCKER ENERGY, hydrogen bond acceptors (HBAs) and donors (HBAs); and Sybyl-X 2.0 for electronegativity*.

### Molecular Docking Studies

**FIGURE 6:**
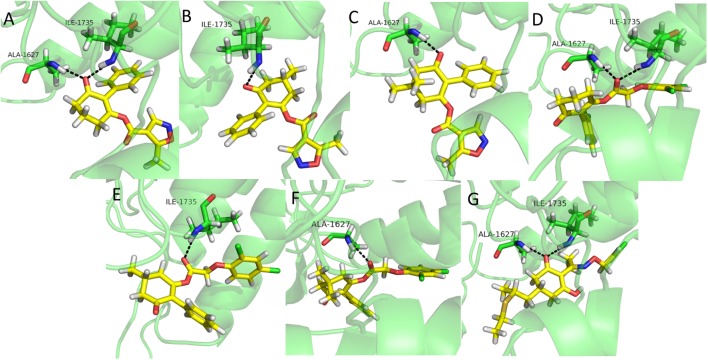
Binding mode of compounds 3a **(A)**, 3b **(B)**, 3c **(C)**, 3d **(D)**, 3e **(E)**, 3f **(F)**, and clethodim **(G)** to ACCase in the active site. Black dashed line represents the hydrogen bond.

Molecular docking is a perfect method for predicting the interactions between small molecules and the receptor binding cavity at the molecular level (Mckay et al., 2012; Fu et al., 2017; Zhang et al., 2018). In order to understand the mechanism by which the synthesized compounds acted on the target site of ACCase, molecular docking experiments were carried out. The results showed that compounds **3a**, **3d**, and clethodim had similar interactions with the surrounding amino acid residues. The keto-carbonyl of **3a** and the ester-carbonyl of **3d** appeared two hydrogen bonds in the active pocket, respectively, which effectively increased the stability of ligand binding to ACCase (Figures 6A,D). Similarly, the keto-carbonyl of clethodim also formed two hydrogen bonds with Ala1627 and ILE1735 (Figure 6G). However, the keto-carbonyl of **3b** and the ester-carbonyl of **3e** formed one hydrogen bond with ILE1735, respectively (Figures 6B,E), while the keto-carbonyl of **3c** and the ester-carbonyl of **3f** generated one hydrogen bond with Ala1627, respectively (Figures 6C,F). The distances from the oxygen atom to these two residues of clethodim were 2.2 and 2.0 Å, respectively; the corresponding distances for compound **3d** were 2.0 and 1.9 Å, while those of **3a** were 3.0 and 3.1 Å. The short distances from the oxygen atom to the two binding residues in compound **3d** may have increased the ACCase inhibition (**3d**>clethodim>**3a**).

**FIGURE 7:**
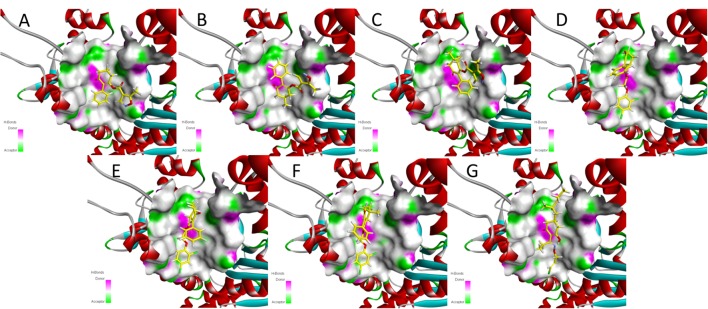
Docking modeling of compounds 3a **(A)**, 3b **(B)**, 3c **(C)**, 3d **(D)**, 3e **(E)**, 3f **(F)**, and clethodim **(G)** to ACCase in the active site. Yellow line represents carbon atoms, gray line represents hydrogen atoms, light yellow line represents sulfur atoms, red line represents oxygen atoms, light blue line represents nitrogen atoms, and green line represents chlorine atoms.

Meanwhile, the docking scores of the synthesized compounds and clethodim were investigated. The values were all positive, indicating that all the synthesized compounds and clethodim achieved an excellent complex with ACCase (Figure 7). Interestingly, it was found that compounds **3a**, **3b**, **3c** had similar binding postures at the active pocket, and the position of the cyclohexanedione ring of **3a** was the same as that of clethodim, which may have led to **3a** forming a better combination with ACCase at the active site than **3b** and **3c** (Figures 7A–C, G). Moreover, similar binding postures for compounds **3d**, **3e**, **3f**, and clethodim were observed at the active site. However, the benzene ring of compound **3d**, attached to the cyclohexanedione, was observed a deflection, which potentially shorten the distance between the ester carbonyl and the amino acid residues, and resulting in compound **3d** having excellent activity similar to that of clethodim (Figures 7D–F). Based on the docking studies and in combination with the bioassay results, compound **3d** may have the same mode of action as clethodim.

## Conclusion

In summary, on the basis of SAR and active subunit combinations, a novel series of 2-phenyl-3-cyclohexanedione enol ester derivatives were designed and synthesized. Most of the target compounds revealed remarkable herbicidal activity, with some even being superior to clethodim. Much to our delight, compounds **3a**, **3d**, and **3e** displayed promising herbicidal activity at a rate of 300 g ai/ha. Moreover, the herbicidal activity of compound **3d** was better than that of clethodim and it was safe for maize. Compound **3d** inhibited ACCase activity significantly, with a value of 0.061 nmol h^-1^ mg^-1^ protein, which was superior to that of clethodim. The molecular docking experiments indicated that the promising herbicide potency could be attributed to the interactions between the synthesized compounds and the surrounding residues, which were similar in compound **3d** and clethodim, and represented an excellent combination with the active pocket of ACCase. The present work indicates that the novel cyclohexanedione skeleton as a potential lead compound for ACCase inhibitor discovery.

## Author Contributions

FYe and YF developed the concept of the work. PM carried out the synthesis. PM and Y-YZ carried out the biological activity tests, molecular docking, and comparisons. PL conducted the ACCase activity assay. FYa performed the mass spectrometric analysis. FYe wrote the paper.

## Conflict of Interest Statement

The authors declare that the research was conducted in the absence of any commercial or financial relationships that could be construed as a potential conflict of interest.

## Supplementary Material

DATA SHEET S1 Electronic Supplementary Material.
