# Supplementary material for: Herbicidal Activity and Molecular Docking Study of Novel ACCase Inhibitors
Source: Front Plant Sci. 2018 Dec 18;9:1850. doi: 10.3389/fpls.2018.01850 (PMC6305411; doi:10.3389/fpls.2018.01850)
Supplement: DATA SHEET S1 — Electronic Supplementary Material. [file Data_Sheet_1.PDF]

Supplementary Material

Herbicidal Activity and Molecular Docking Study of Novel ACCase Inhibitors

Fei Ye[1], Peng Ma[1], Yuan-Yuan Zhang[1], Ping Li[1], Fei Yang[1], Ying Fu [1]*

[1]Department of Applied Chemistry, College of Science, Northeast Agricultural University, Harbin 150030, China

Correspondence author:

Professor Ying Fu, Department of Applied Chemistry, College of Science, Northeast Agricultural University, Harbin 150030, China

Tel: 86-451-55190070

Email address: fuying@neau.edu.cn

Supplementary Information

Compound 2a

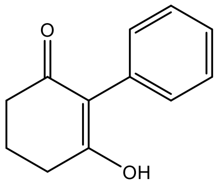

$C_{12}H_{12}O_2$

IR

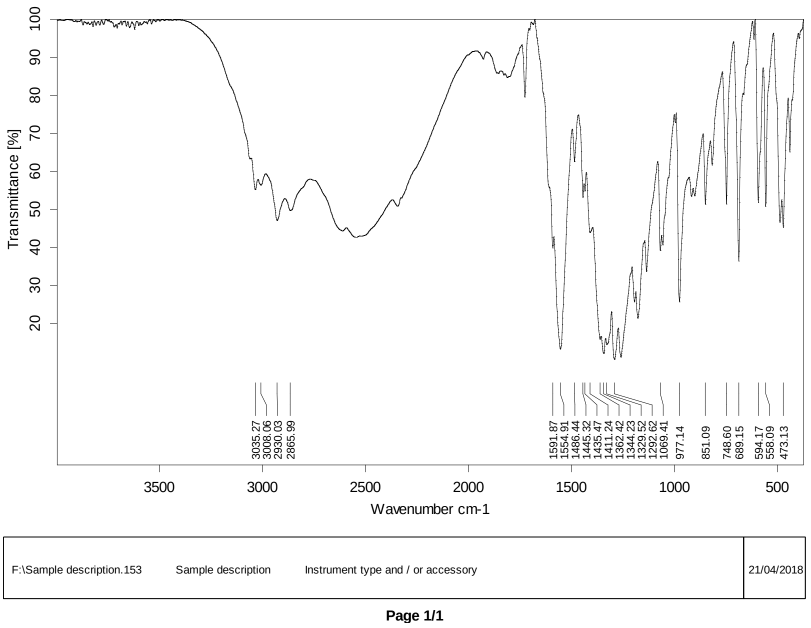

^{1}H NMR

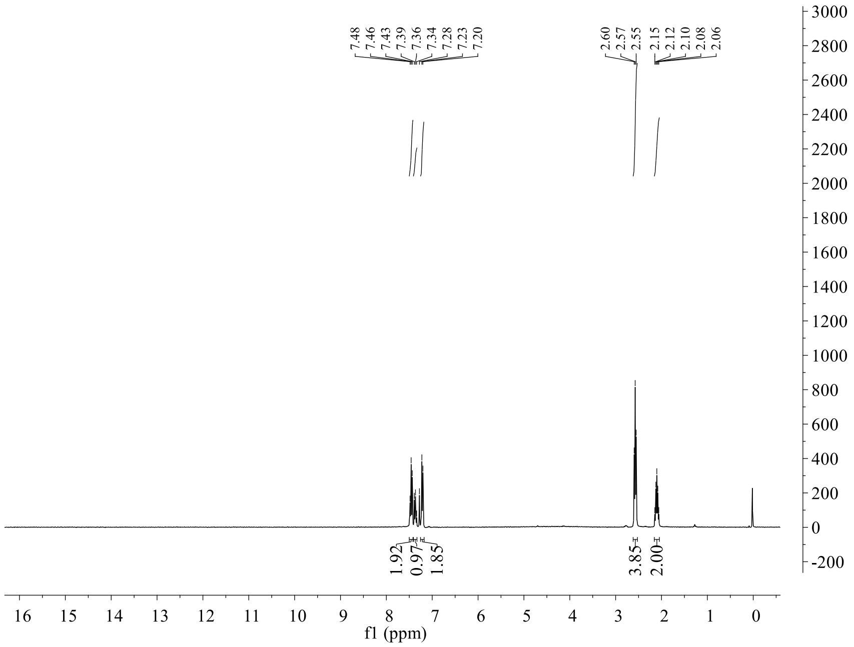

^{13}C NMR

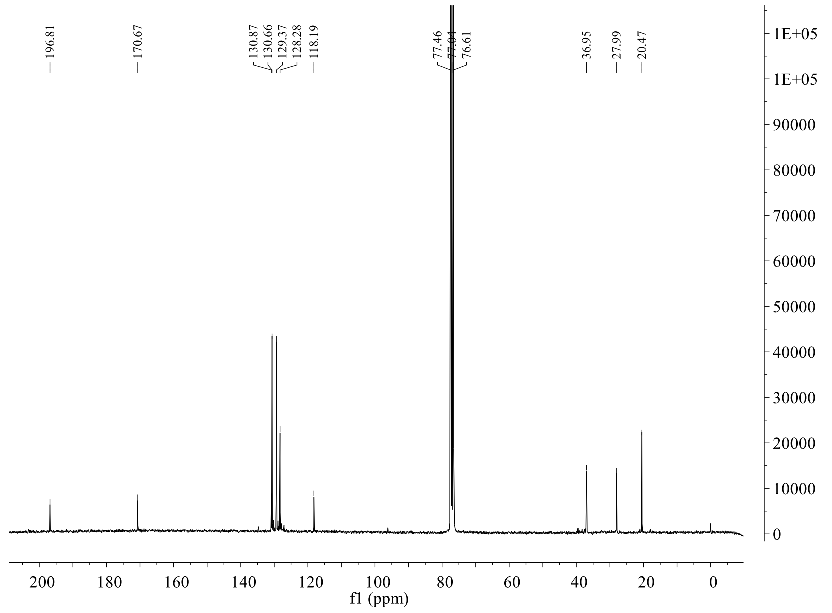

196.81
170.67
130.87
130.66
129.37
128.28
118.19
77.46
77.04
76.61
36.95
27.99
20.47
1E+05
1E+05
90000
80000
70000
60000
50000
40000
30000
20000
10000
0
200
180
160
140
120
100
80
60
40
20
0
f1 (ppm)

Compound 2b

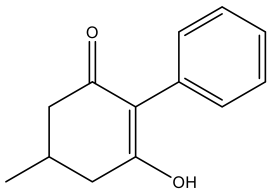

$C_{13}H_{14}O_2$

IR

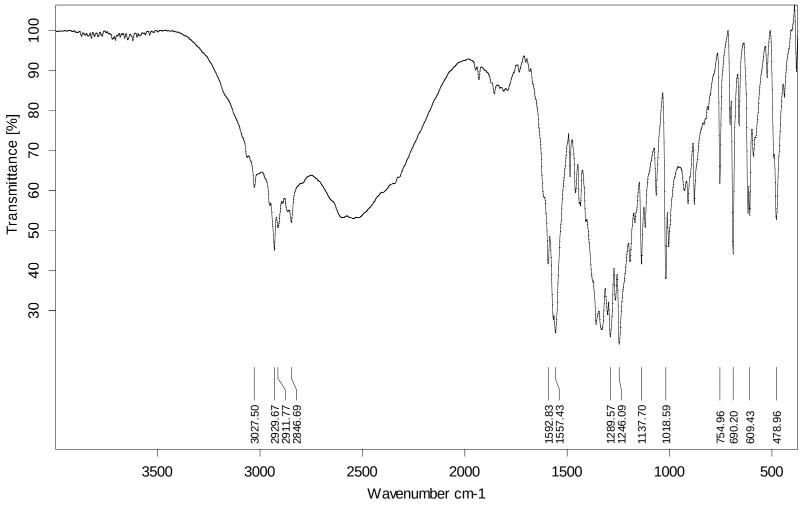

^{1}H NMR

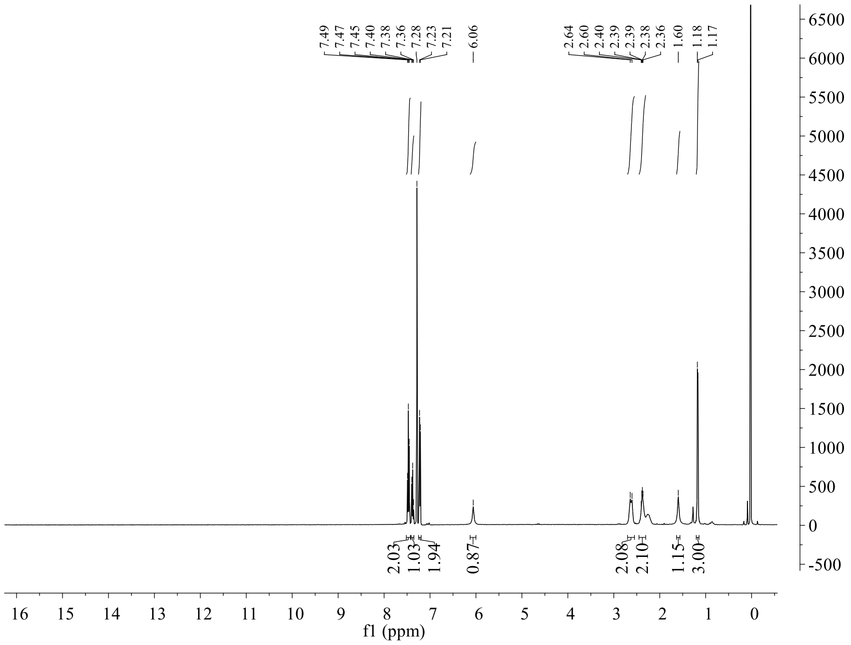

^{13}C NMR

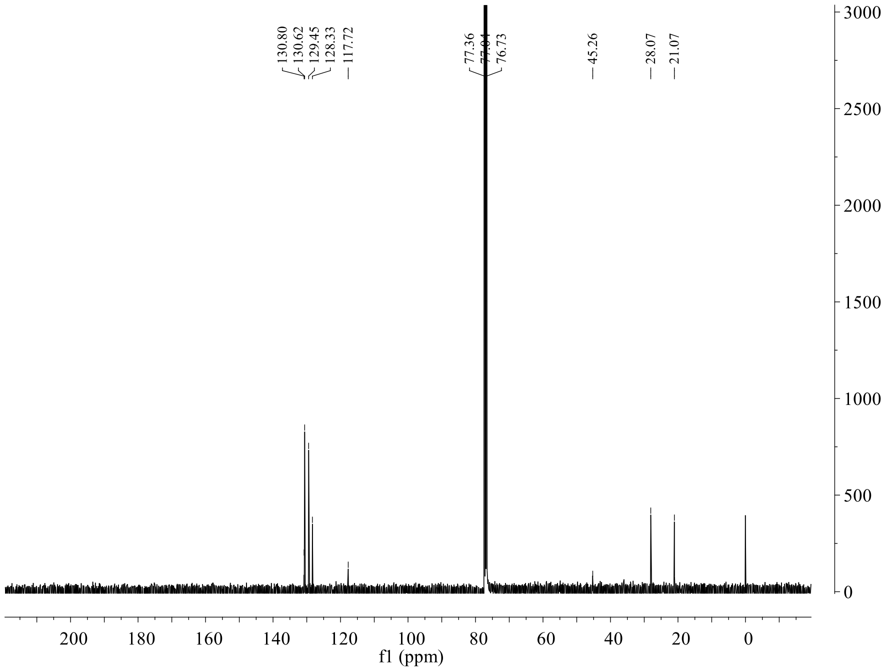
130.80
130.62
129.45
128.33
117.72
77.36
77.04
76.73
45.26
28.07
21.07
3000
2500
2000
1500
1000
500
0
200
180
160
140
120
100
80
60
40
20
0
f1 (ppm)

Compound 2c

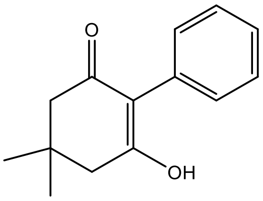

$C_{14}H_{16}O_2$

IR

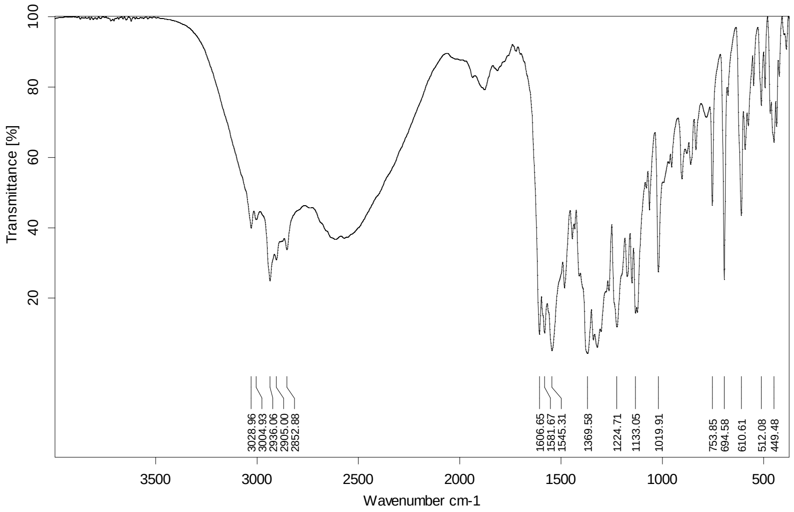

¹H NMR

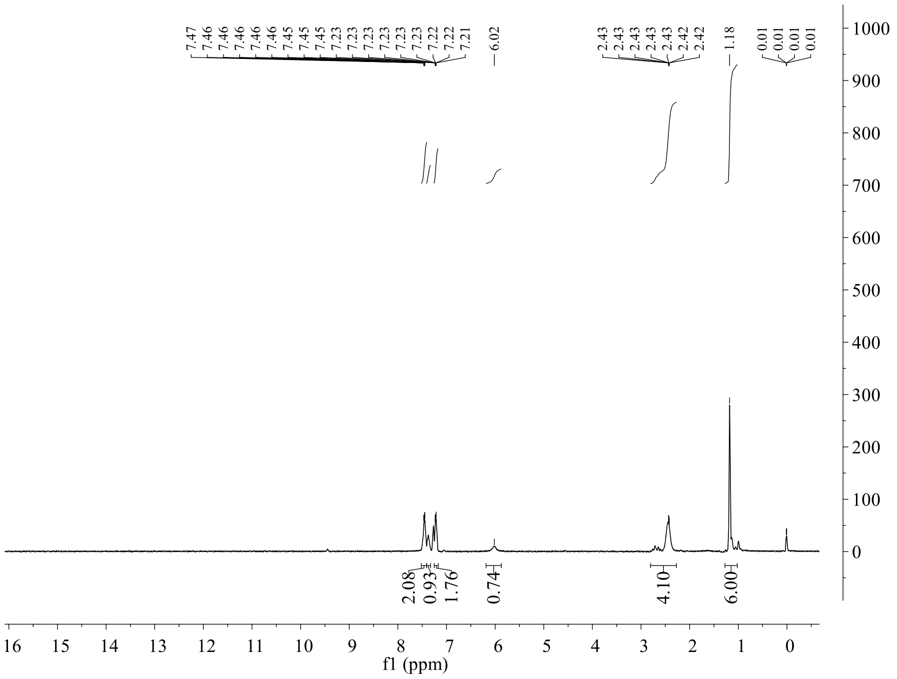

^{13}C NMR

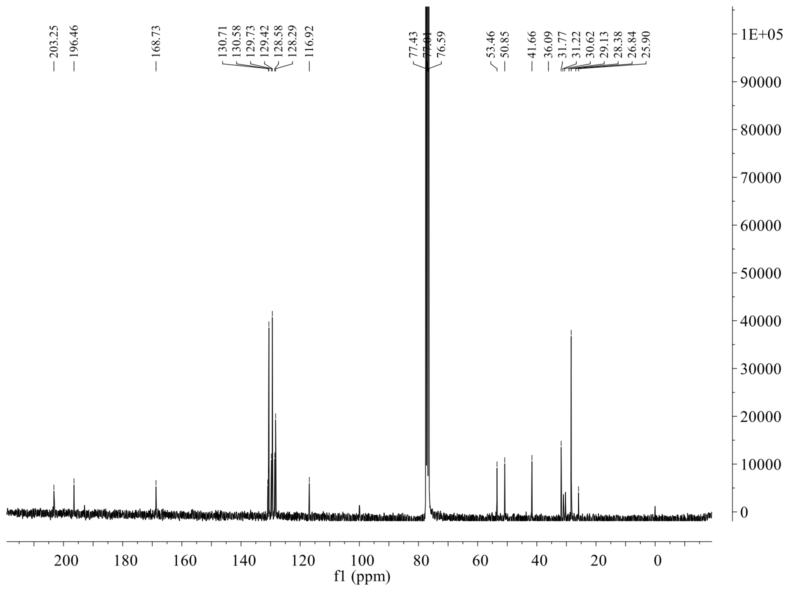
203.25
196.46
168.73
130.71
130.58
129.73
129.42
128.58
128.29
116.92
77.43
77.01
76.59
53.46
50.85
41.66
36.09
31.77
31.22
30.62
29.13
28.38
26.84
25.90
1E+05
90000
80000
70000
60000
50000
40000
30000
20000
10000
0
200
180
160
140
120
100
80
60
40
20
0
f1 (ppm)

Compound 3a

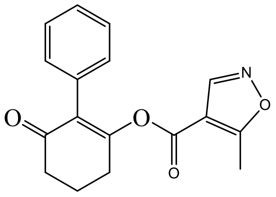

$C_{17}H_{15}NO_4$

IR

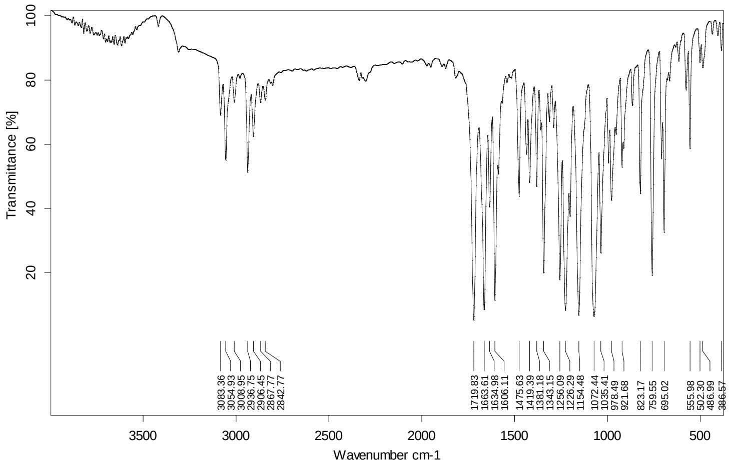

^{1}H NMR

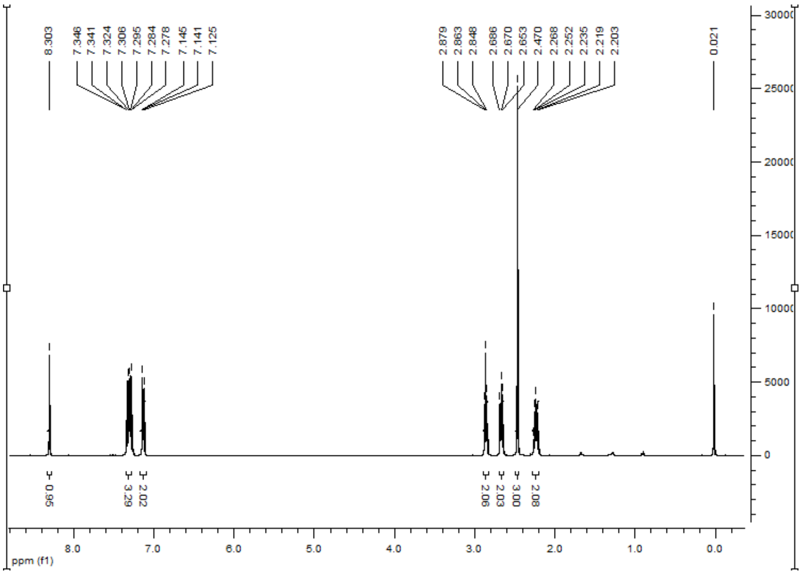

^{13}C NMR

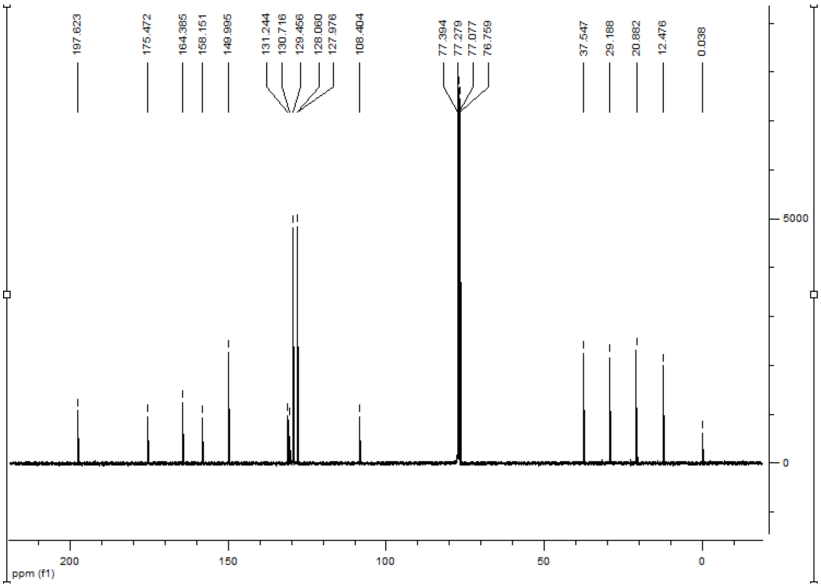
197.623
175.472
164.385
158.151
149.995
131.244
130.716
129.456
128.060
127.976
108.404
77.394
77.279
77.077
76.759
37.547
29.188
20.882
12.476
0.038
5000
0
200
150
100
50
0
ppm (f1)

HRMS

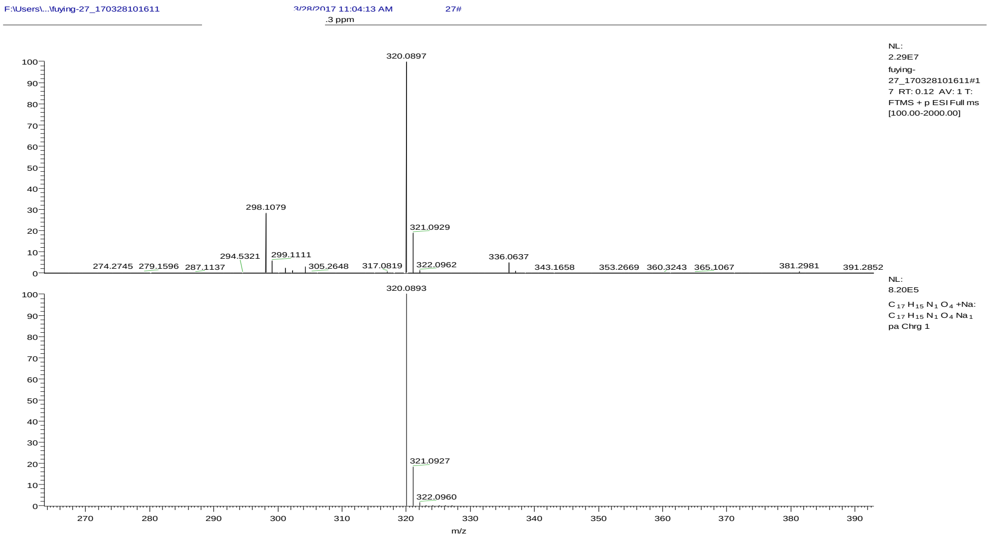
F:\Users\...\fuying-27_170328101611
3/28/2017 11:04:13 AM
27#
.3 ppm
NL:
2.29E7
fuying-27_170328101611#17 RT: 0.12 AV: 1 T: FTMS + p ESI Full ms [100.00-2000.00]
320.0897
298.1079
321.0929
294.5321
299.1111
336.0637
274.2745
279.1596
287.1137
305.2648
317.0819
322.0962
343.1658
353.2669
360.3243
365.1067
381.2981
391.2852
NL:
8.20E5
C 17 H 15 N 1 O 4 +Na:
C 17 H 15 N 1 O 4 Na 1
pa Chrg 1
320.0893
321.0927
322.0960
m/z

Compound 3b

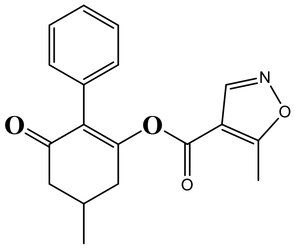

$C_{18}H_{17}NO_4$

IR

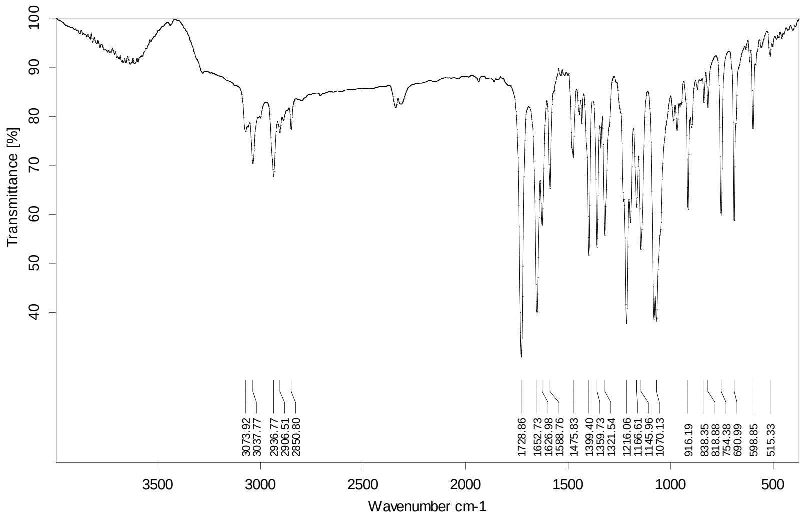

^{1}H NMR

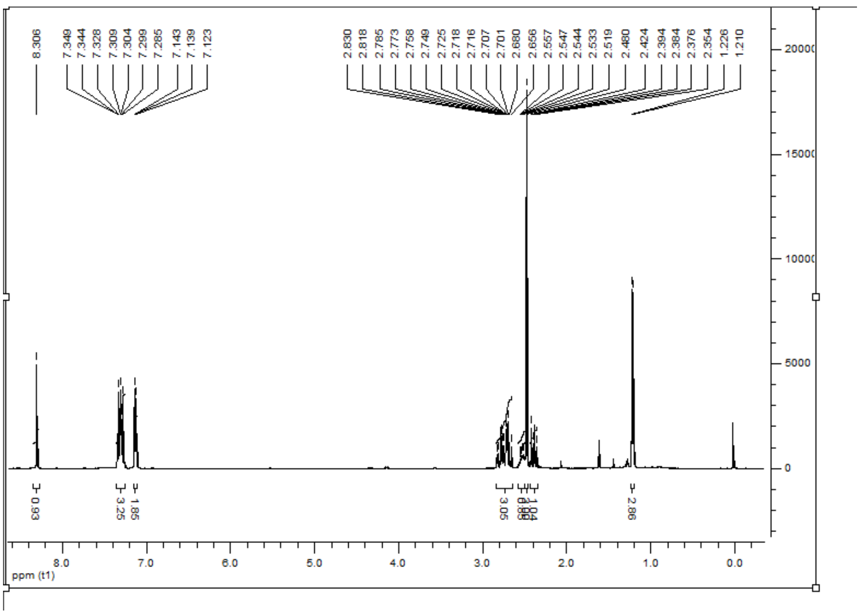

^{13}C NMR

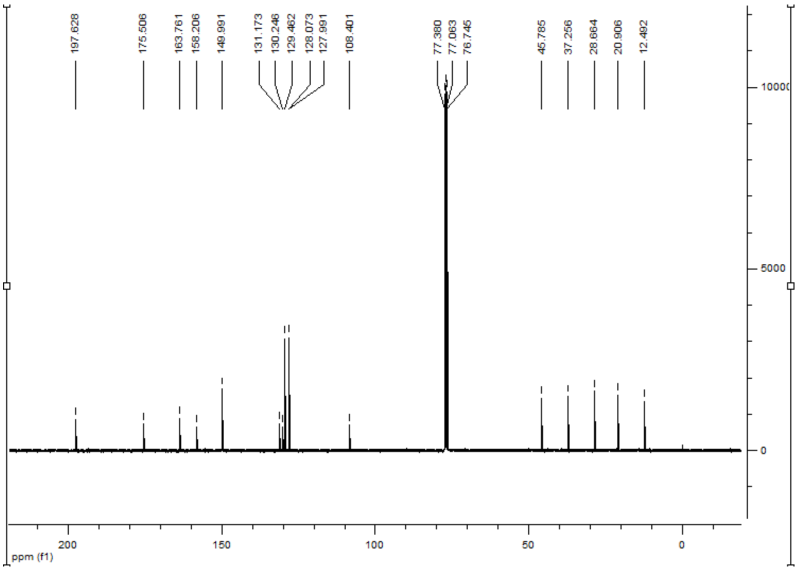
197.628
175.506
163.761
158.206
149.991
131.173
130.246
129.462
128.073
127.991
108.401
77.380
77.063
76.745
45.785
37.256
28.664
20.906
12.492
10000
5000
0
200
150
100
50
0
ppm (f1)

HRMS

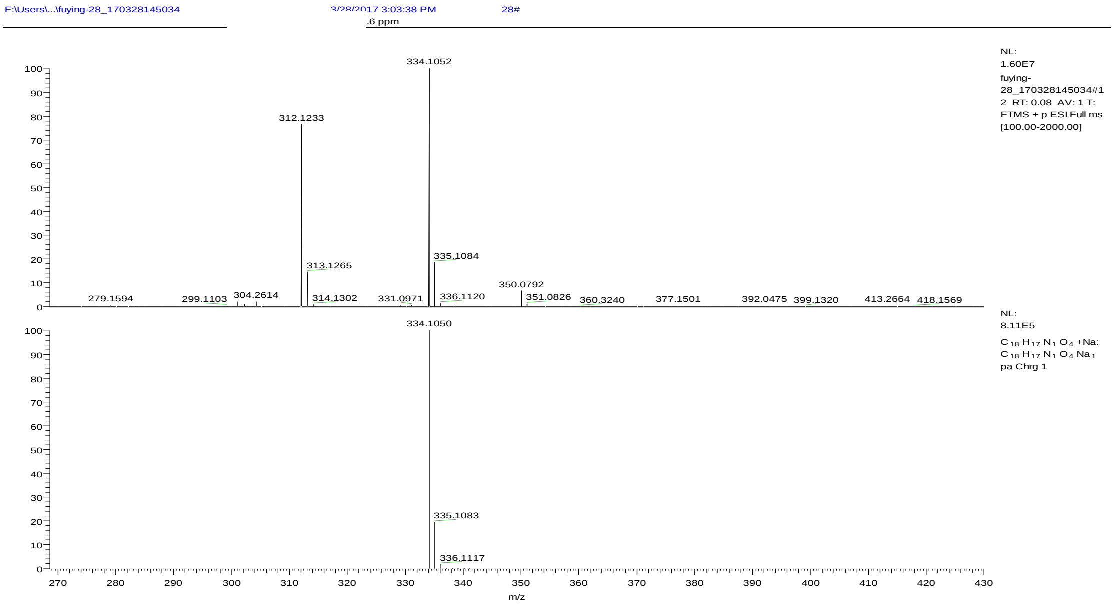
F:\Users\...\fuying-28_170328145034
3/28/2017 3:03:38 PM
28#
.6 ppm
NL:
1.60E7
fuying-28_170328145034#1
2 RT: 0.08 AV: 1 T:
FTMS + p ESI Full ms
[100.00-2000.00]
334.1052
312.1233
313.1265
335.1084
350.0792
279.1594
299.1103
304.2614
314.1302
331.0971
336.1120
351.0826
360.3240
377.1501
392.0475
399.1320
413.2664
418.1569
NL:
8.11E5
C 18 H 17 N 1 O 4 +Na:
C 18 H 17 N 1 O 4 Na 1
pa Chrg 1
334.1050
335.1083
336.1117
m/z

Compound 3c

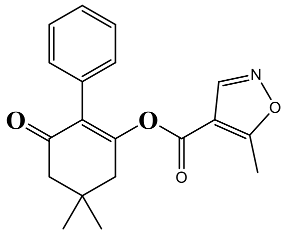

$C_{19}H_{19}NO_4$

IR

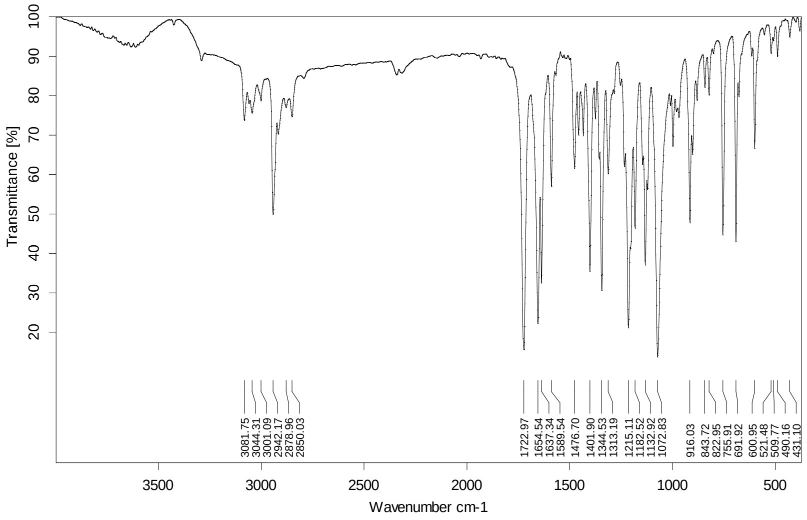

^{1}H NMR

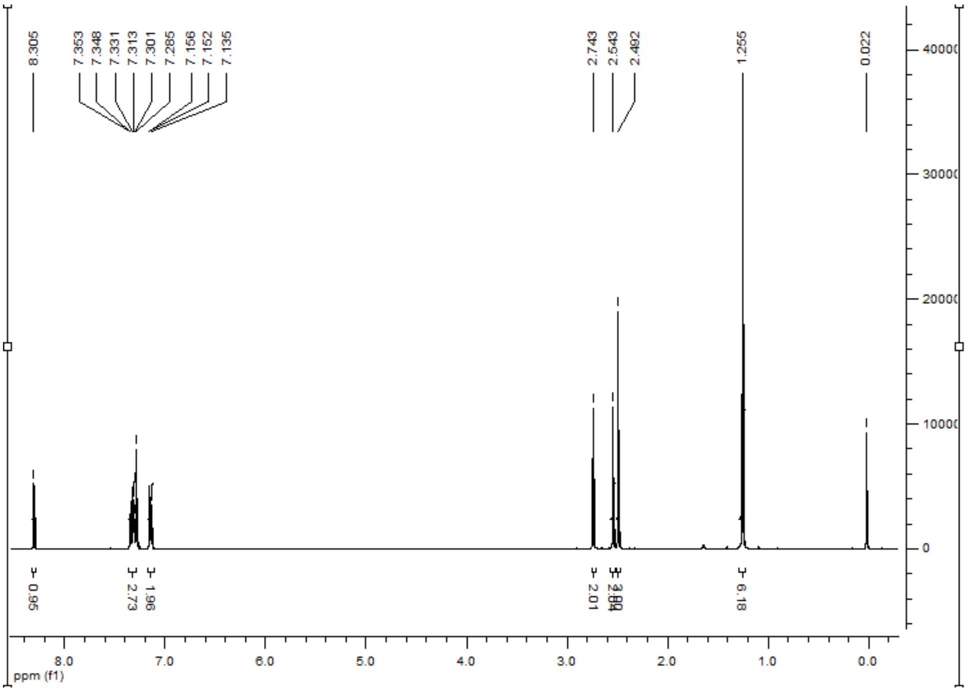

^{13}C NMR

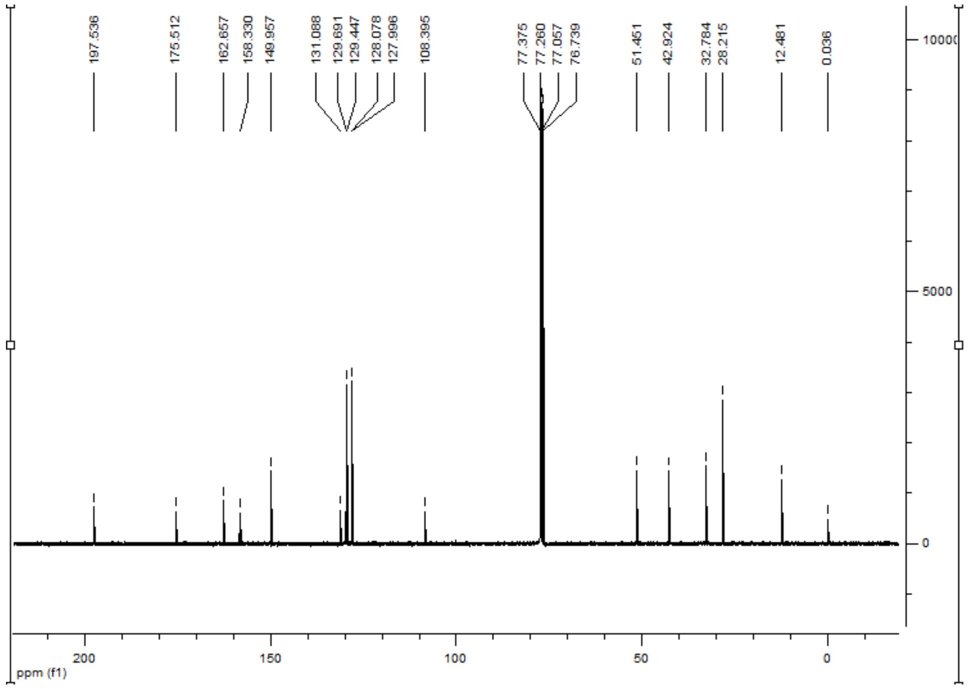
197.536
175.512
162.657
158.330
149.957
131.088
129.691
129.447
128.078
127.996
108.395
77.375
77.260
77.057
76.739
51.451
42.924
32.784
28.215
12.481
0.036
10000
5000
0
200
150
100
50
0
ppm (f1)

HRMS

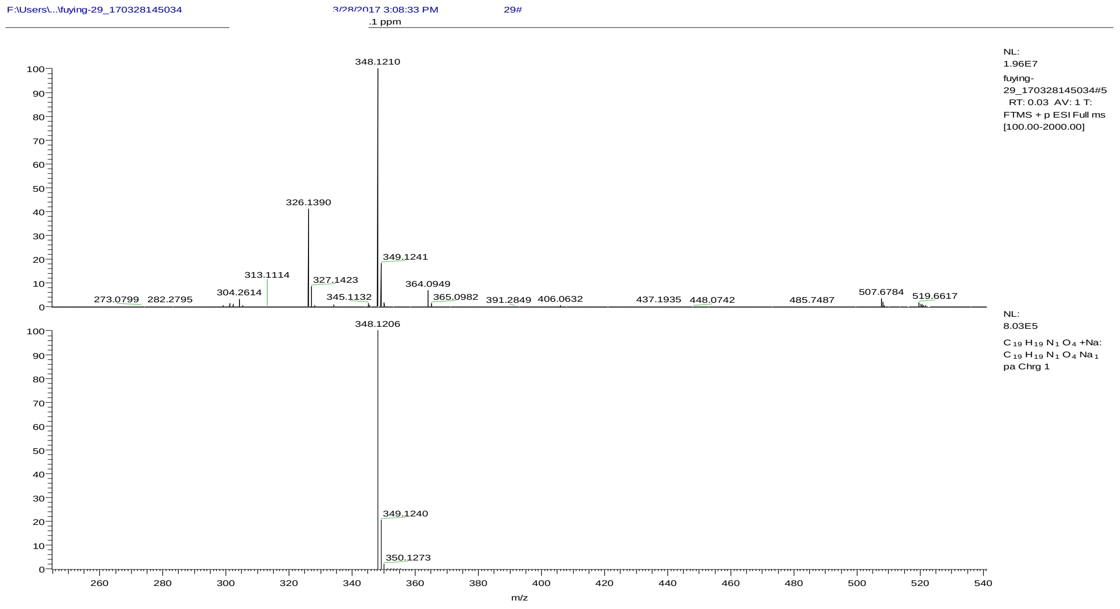
F:\Users\...\fuying-29_170328145034
3/28/2017 3:08:33 PM
29#
.1 ppm
NL:
1.96E7
fuying-29_170328145034#5
RT: 0.03 AV: 1 T: FTMS + p ESI Full ms [100.00-2000.00]
348.1210
326.1390
349.1241
313.1114
327.1423
364.0949
304.2614
345.1132
365.0982
273.0799
282.2795
391.2849
406.0632
437.1935
448.0742
485.7487
507.6784
519.6617
NL:
8.03E5
C 19 H 19 N 1 O 4 +Na:
C 19 H 19 N 1 O 4 Na 1
pa Chrg 1
348.1206
349.1240
350.1273
m/z

Compound 3d

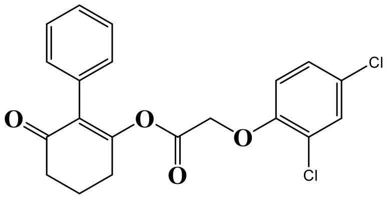

$C_{20}H_{16}Cl_2O_4$

IR

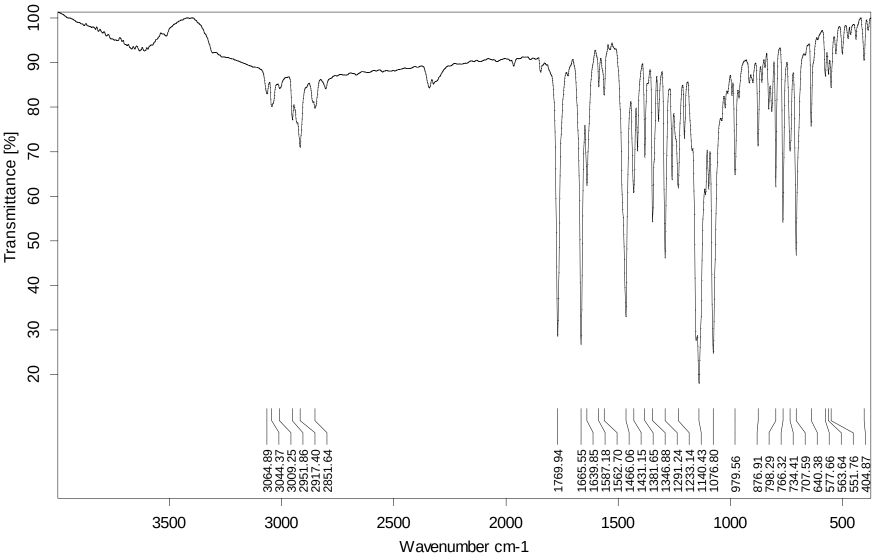

^{1}H NMR

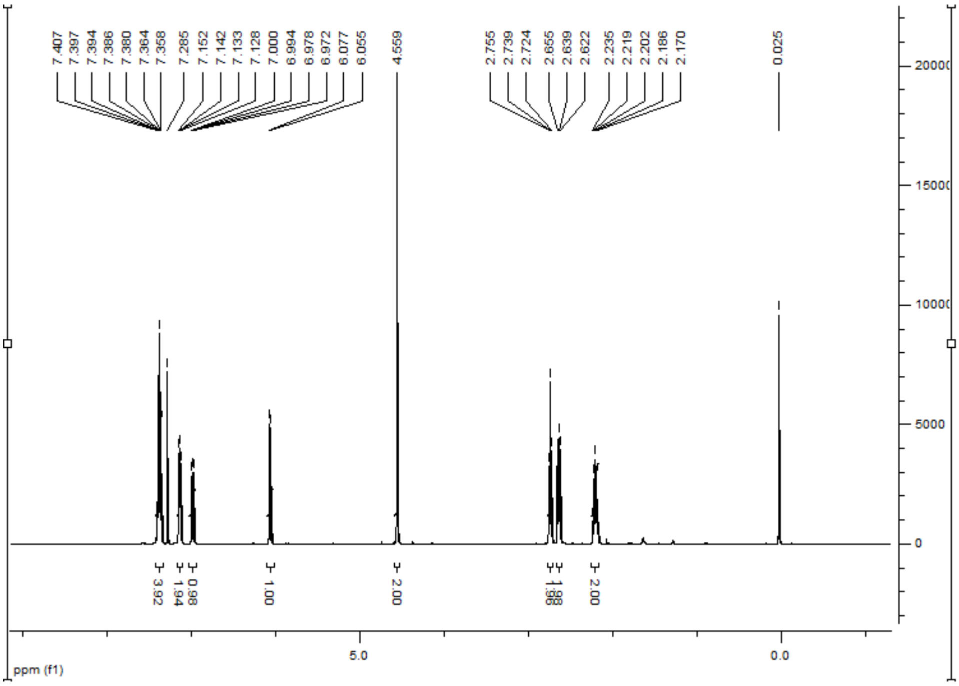

^{13}C NMR

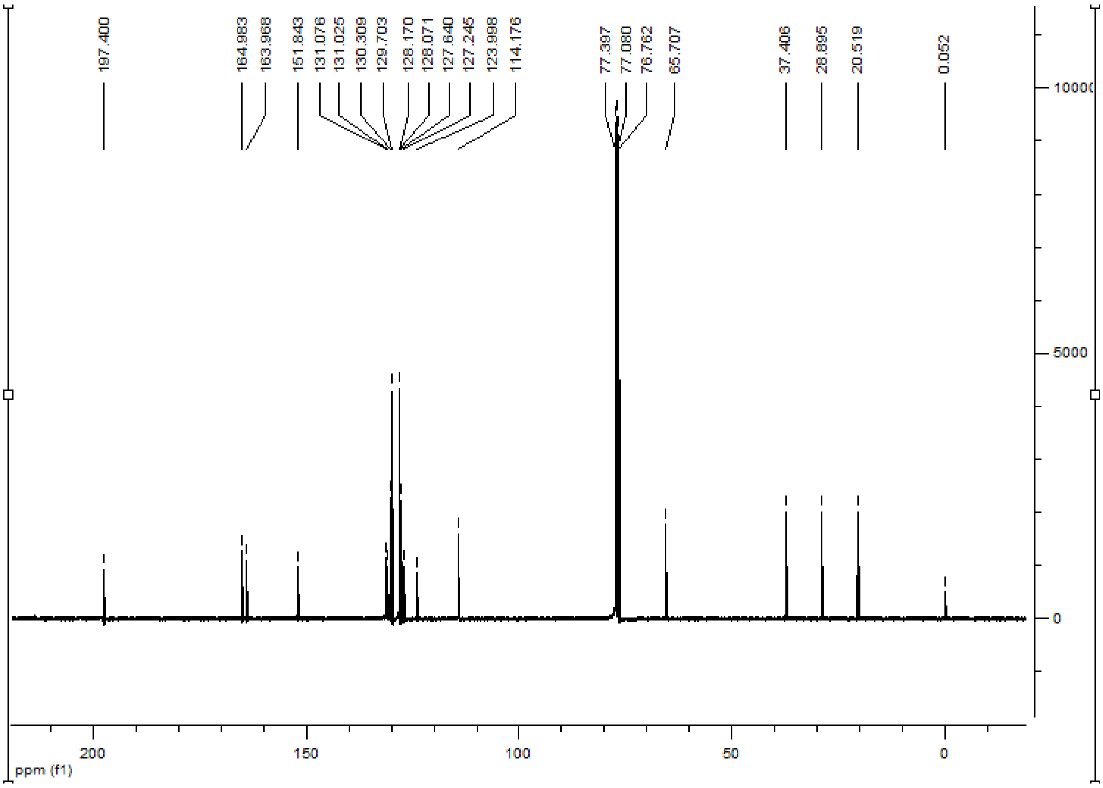
197.400
164.983
163.968
151.843
131.076
131.025
130.309
129.703
128.170
128.071
127.640
127.245
123.998
114.176
77.397
77.080
76.762
65.707
37.406
28.895
20.519
0.052
10000
5000
0
200
150
100
50
0
ppm (f1)

HRMS

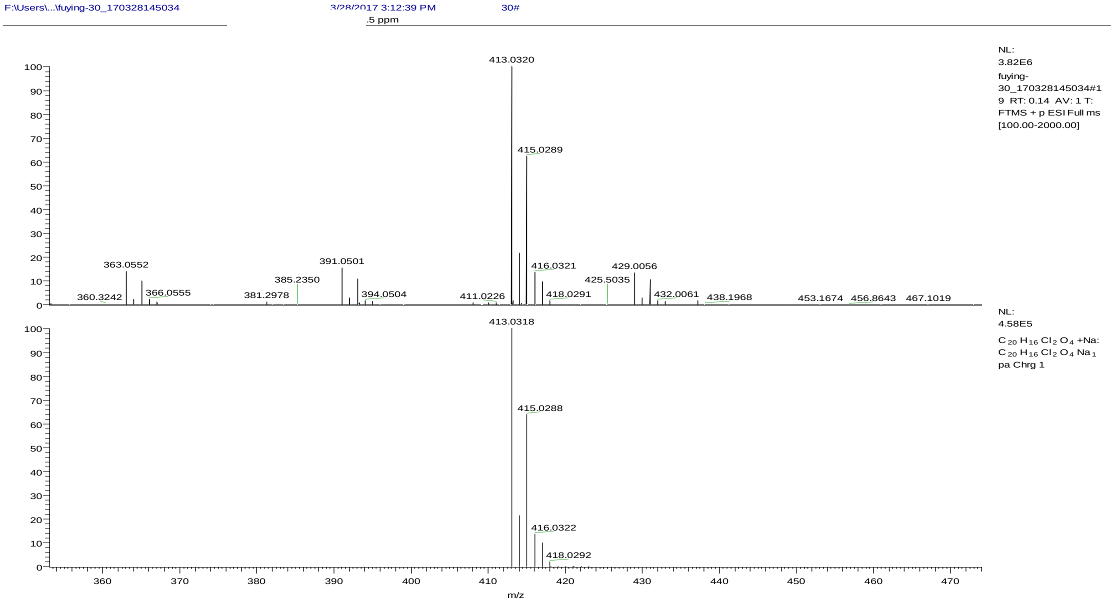
413.0320
415.0289
363.0552
391.0501
385.2350
416.0321
429.0056
425.5035
360.3242
366.0555
381.2978
394.0504
411.0226
418.0291
432.0061
438.1968
453.1674
456.8643
467.1019
NL: 3.82E6
FTMS + p ESI Full ms [100.00-2000.00]
413.0318
415.0288
416.0322
418.0292
NL: 4.58E5
C 20 H 16 Cl 2 O 4 +Na:
C 20 H 16 Cl 2 O 4 Na 1
pa Chrg 1
m/z

Compound 3e

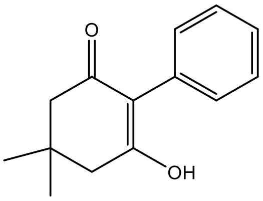

$C_{14}H_{16}O_2$

IR

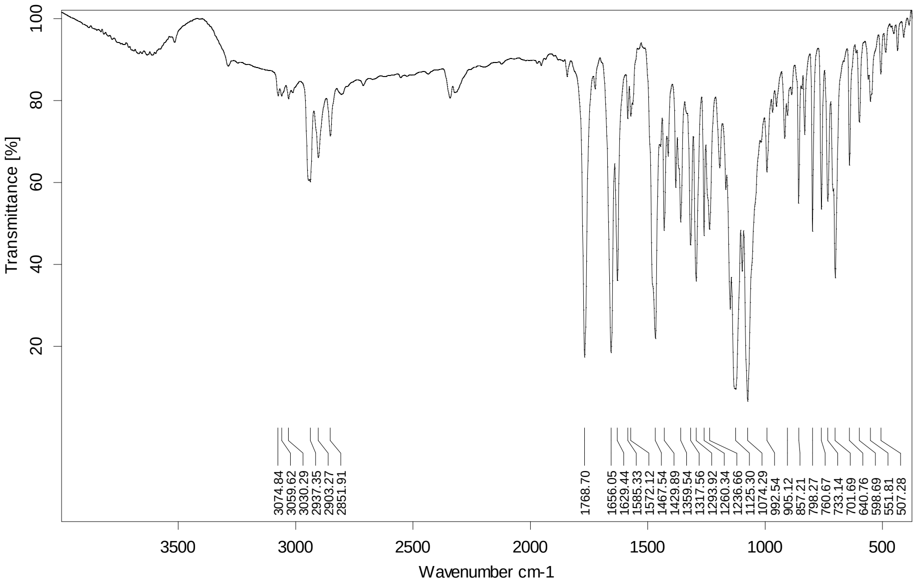

^{1}H NMR

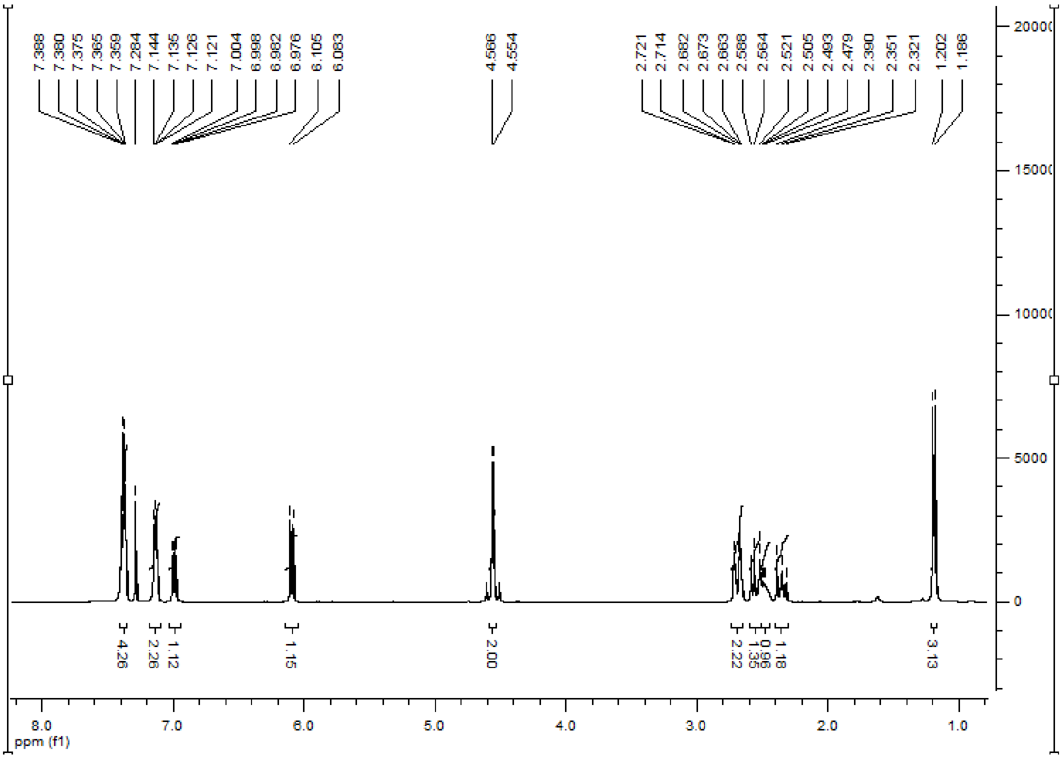

^{13}C NMR

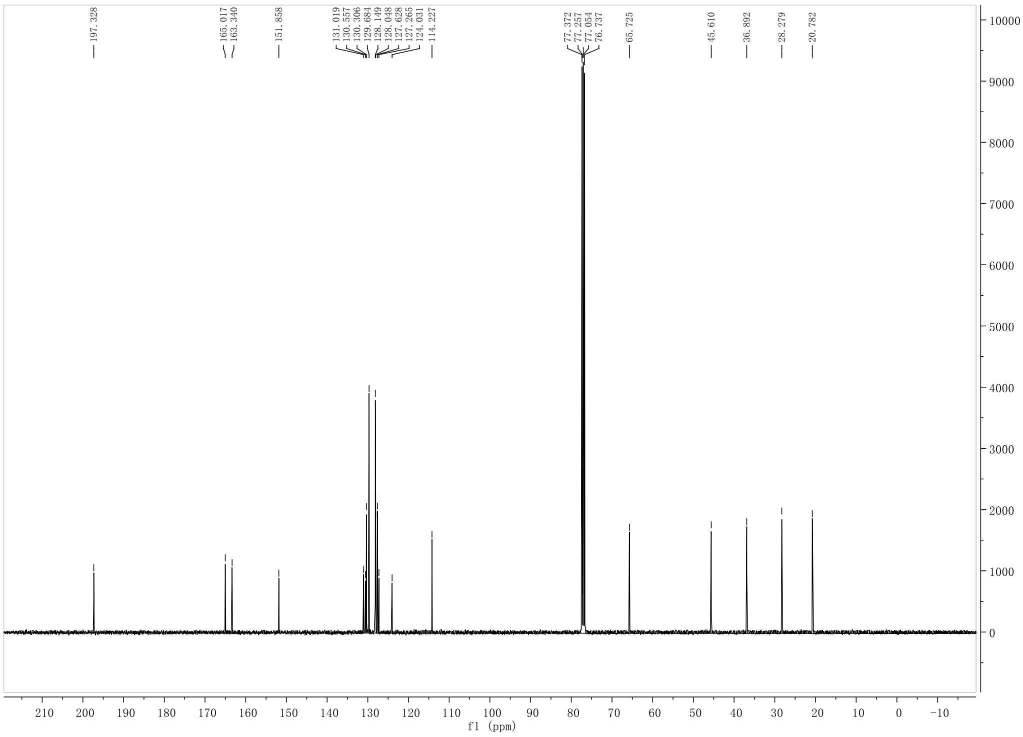

197.328
165.017
163.340
151.858
131.019
130.557
130.306
129.684
128.149
128.048
127.628
127.265
124.031
114.227
77.372
77.257
77.054
76.737
65.725
45.610
36.892
28.279
20.782
f1 (ppm)

HRMS

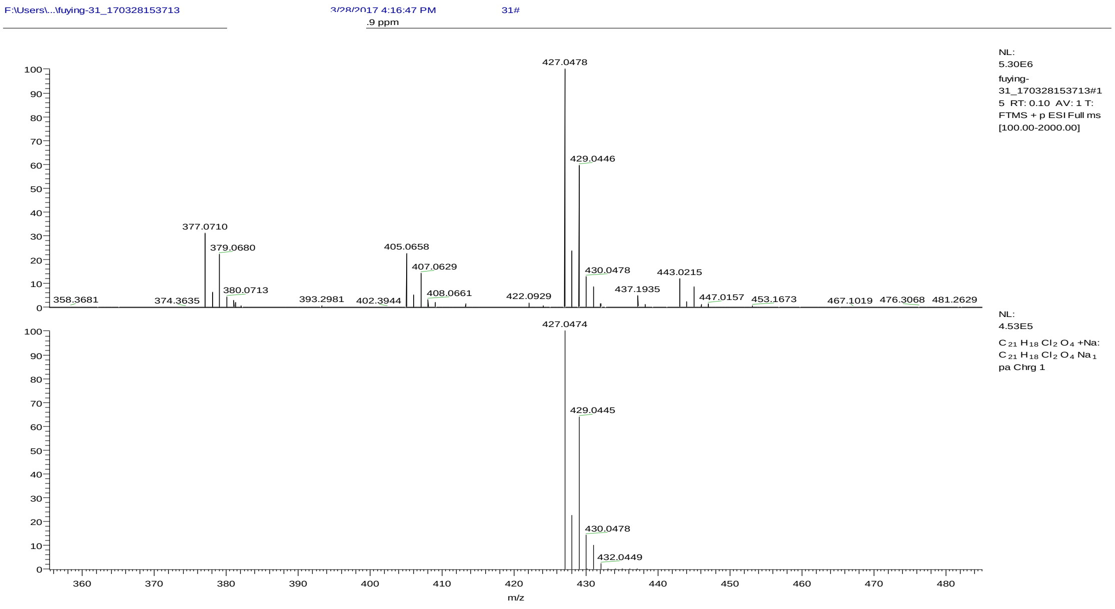

F:\Users\...\fuying-31_170328153713
3/28/2017 4:16:47 PM
31#
.9 ppm
NL: 5.30E6
fuying-31_170328153713#15 RT: 0.10 AV: 1 T: FTMS + p ESI Full ms [100.00-2000.00]
427.0478
429.0446
377.0710
379.0680
405.0658
407.0629
430.0478
443.0215
437.1935
380.0713
408.0661
358.3681
374.3635
393.2981
402.3944
422.0929
447.0157
453.1673
467.1019
476.3068
481.2629
NL: 4.53E5
C 21 H 18 Cl 2 O 4 +Na: C 21 H 18 Cl 2 O 4 Na 1 pa Chrg 1
427.0474
429.0445
430.0478
432.0449
m/z

Compound 3f

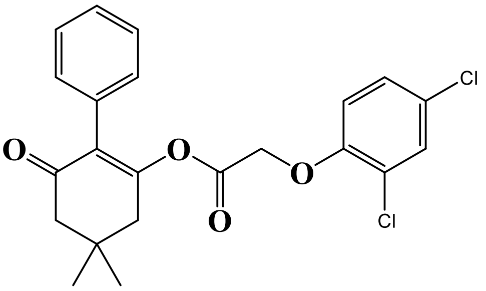

$C_{22}H_{20}Cl_2O_4$

IR

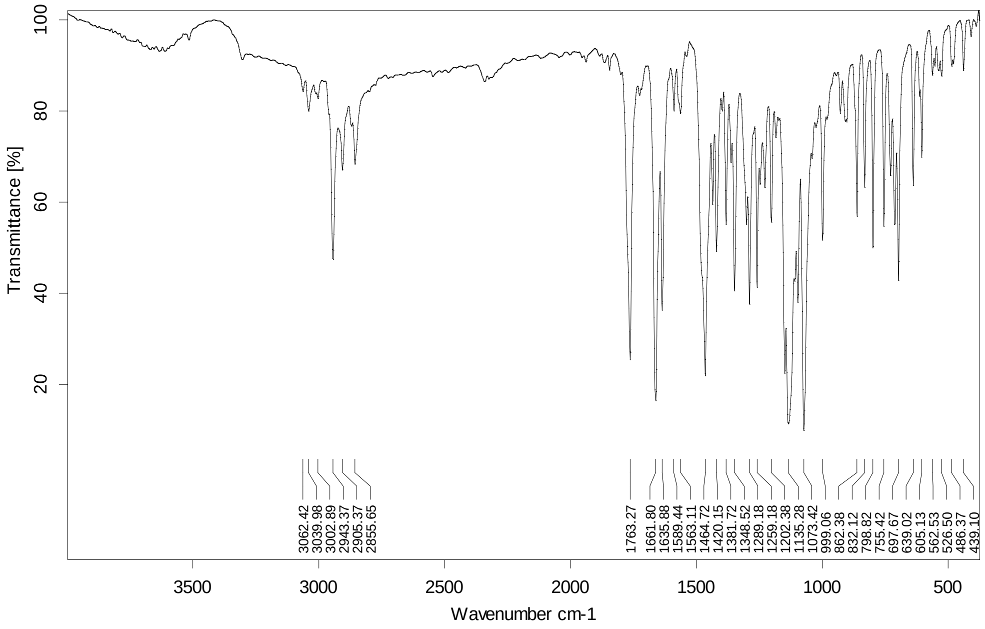

^{1}H NMR

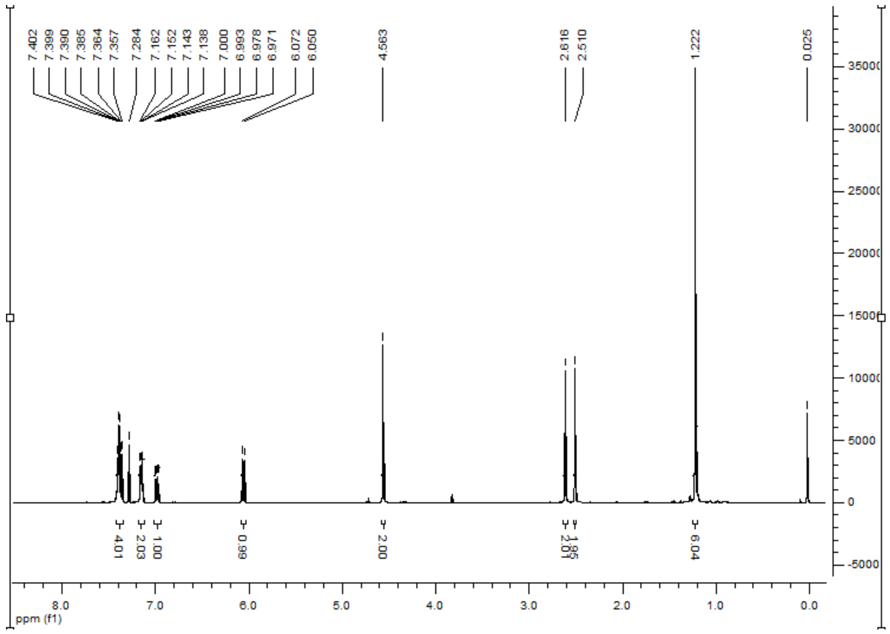

^{13}C NMR

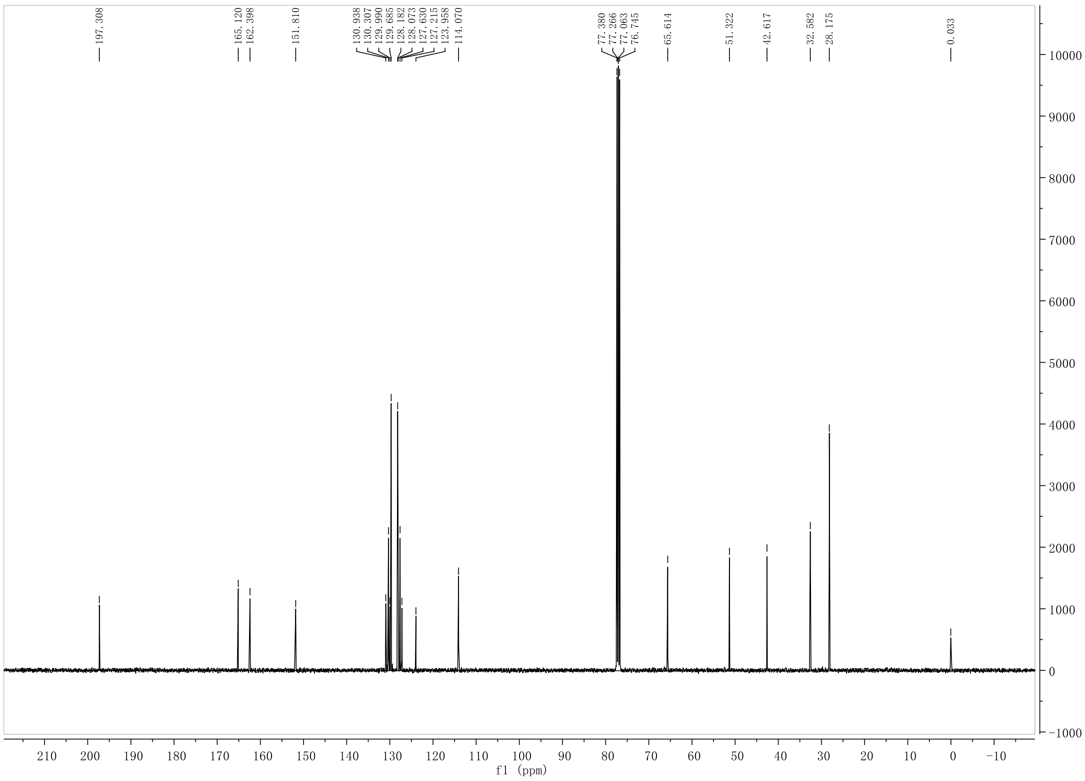
197.308
165.120
162.398
151.810
130.938
130.307
129.990
129.685
128.182
128.073
127.630
127.215
123.958
114.070
77.380
77.266
77.063
76.745
65.614
51.322
42.617
32.582
28.175
0.033
f1 (ppm)

HRMS

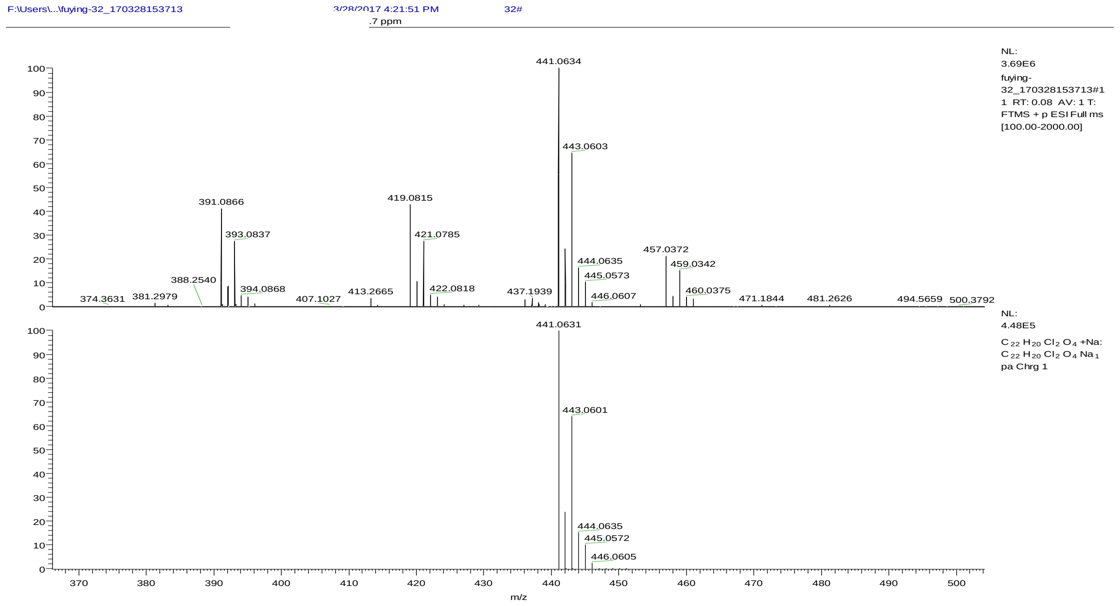
F:\Users\...\fuying-32_170328153713
3/28/2017 4:21:51 PM
32#
NL: 3.69E6
fuying-32_170328153713#1 RT: 0.08 AV: 1 T: FTMS + p ESI Full ms [100.00-2000.00]
441.0634
443.0603
391.0866
419.0815
393.0837
421.0785
457.0372
459.0342
444.0635
445.0573
388.2540
394.0868
422.0818
460.0375
374.3631
381.2979
407.1027
413.2665
437.1939
446.0607
471.1844
481.2626
494.5659
500.3792
NL: 4.48E5
C 22 H 20 Cl 2 O 4 +Na: C 22 H 20 Cl 2 O 4 Na 1 pa Chrg 1
441.0631
443.0601
444.0635
445.0572
446.0605
m/z
